# In vivo sonic hedgehog pathway antagonism temporarily results in ancestral proto-feather-like structures in the chicken

**DOI:** 10.1371/journal.pbio.3003061

**Published:** 2025-03-20

**Authors:** Rory L. Cooper, Michel C. Milinkovitch

**Affiliations:** 1 Laboratory of Artificial and Natural Evolution (LANE), Department of Genetics and Evolution, University of Geneva, Geneva, Switzerland; 2 Swiss Institute of Bioinformatics (SIB), Geneva, Switzerland; California Institute of Technology, UNITED STATES OF AMERICA

## Abstract

The morphological intricacies of avian feathers make them an ideal model for investigating embryonic patterning and morphogenesis. In particular, the sonic hedgehog (Shh) pathway is an important mediator of feather outgrowth and branching. However, functional in vivo evidence regarding its role during feather development remains limited. Here, we demonstrate that an intravenous injection of sonidegib, a potent Shh pathway inhibitor, at embryonic day 9 (E9) temporarily produces striped domains (instead of spots) of *Shh* expression in the skin, arrests morphogenesis, and results in unbranched and non-invaginated feather buds—akin to proto-feathers—in embryos until E14. Although feather morphogenesis partially recovers, hatched treated chickens exhibit naked skin regions with perturbed follicles. Remarkably, these follicles are subsequently reactivated by seven weeks post-hatching. Our RNA-sequencing data and rescue experiment using Shh-agonism confirm that sonidegib specifically down-regulates Shh pathway activity. Overall, we provide functional evidence for the role of the Shh pathway in mediating feather morphogenesis and confirm its role in the evolutionary emergence and diversification of feathers.

## Abstract 2

The Sonic Hedgehog (Shh) pathway is a key regulator of feather development. These authors show that in vivo Shh inhibition during early chicken embryogenesis temporarily results in unbranched and non-invaginated embryonic feather buds, similar to those seen in some early dinosaur ancestors of birds.

## Introduction

**Fig 1:**
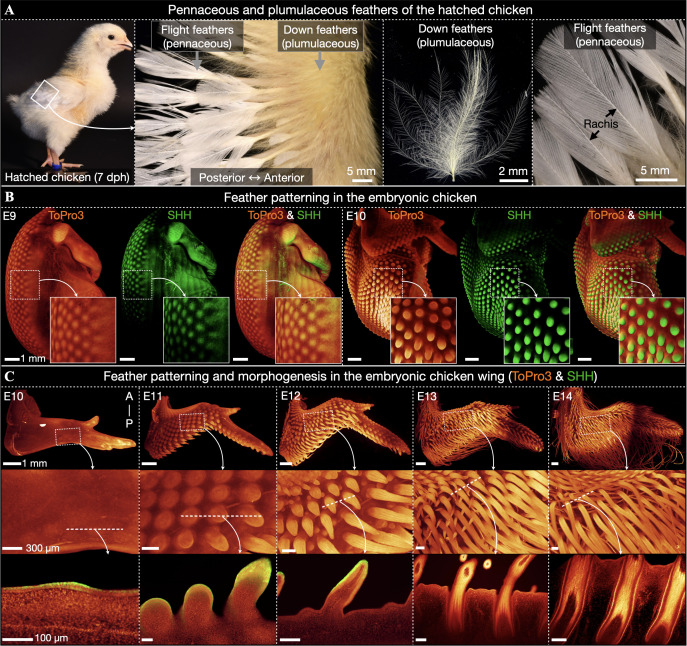
Feather development in embryonic chicken. (**A**) Hatched chickens at seven day post-hatching (7 dph) exhibit both pennaceous (with a central rachis) and plumulaceous (without a rachis) feather types. (**B**) At E9, LSFM reveals feather placodes covering most of the dorsum and expressing SHH protein (visualized by immunofluorescence). By E10, these placodes undergo considerable outgrowth and SHH becomes localized to the posterior tip of developing units. (**C**) Feather placodes first emerge on the posterior edge of the wings at E10, before propagating to cover the entire wing by E11. Optical sections reveal epidermal SHH immunofluorescence from E10 to E12. At E12, the longitudinal barbs associated with branching morphogenesis are visible, and by E13, invagination of the follicle is well-advanced. These units continue to develop until E14, at which point feather buds are elongated, keratinized, and exhibit a longitudinal pattern of cell density corresponding to the future feather barbs.

Avian feathers are intricate integumentary appendages, the forms of which vary substantially among species, across body areas, and between juvenile and adult stages. Understanding both the developmental and evolutionary mechanisms underpinning this morphological diversity has long fascinated biologists [1,2]. Although their evolutionary origin has been widely debated, feathers likely first appeared as simple cylindrical monofilaments in the common archosaurian ancestor of dinosaurs and pterosaurs during the Early Triassic [3–5]. Gradually, the morphology of these so-called ‘proto-feathers’ then increased in complexity to first produce simple plumulaceous down-type feathers (which lack a central shaft known as a rachis) and, later, to produce the more highly ordered pennaceous feathers [1,2], including bilaterally symmetric contour feathers and bilaterally asymmetric flight feathers (Fig 1A) [6]. This diversification was necessarily driven by modifications to the spatial expression of conserved developmental genes mediating feather morphogenesis, including the longitudinal domains of sonic hedgehog (*Shh*) expression observed during feather barb formation [7,8]. However, the precise effects of perturbing such molecular signaling throughout feather morphogenesis remain to be comprehensively investigated in vivo. More generally, feathers provide an ideal model for investigating molecular developmental patterning and morphogenesis [6,9].

Although feathers arguably constitute the most complex and highly ordered group of integumentary appendages, their early embryonic development shares broad similarities in molecular signaling and morphogenesis with hairs and scales [10–13]. The morphological development of feathers begins with the emergence of an anatomical placode [12,14]—a local epidermal thickening and an underlying aggregation of dermal cells—associated with conserved patterns of gene expression [9]. Indeed, conserved placode signaling constitutes the foundation of integumentary appendages across diverse vertebrate clades, from the scales of sharks to the hairs of mammals [10–13]—with the exception of crocodile head scales [15,16]. The spatial distribution of placodes is widely considered to be mediated through paradigmatic chemical Turing reaction-diffusion dynamics, in which interactions between diffusing activatory and inhibitory morphogens give rise to stable periodic patterns [17–23]. Research regarding feather propagation in the chicken, and scale propagation in snakes, has revealed that chemical reaction-diffusion patterning is coupled with mechanical processes. Indeed, fibroblast growth factor (FGF) signaling triggers the aggregation of dermal cells which mechanically compress the overlying epidermis, thereby initiating subsequent local molecular signaling [23–26]. The outgrowth of feather placodes then gives rise to elongated and cylindrical feather buds, whilst invagination at their base gives rise to the follicle [6]. Finally, the feather bud divides into individual longitudinal filaments known as barbs, a process termed “branching morphogenesis”.

Previous research has also investigated the post-embryonic development of feathers. For example, the establishment of a Wnt3a signaling gradient appears to be required for the development of bilaterally symmetric contour feathers (but not radially symmetric plumulaceous feathers) during post-embryonic development [27]. Furthermore, coordinated adjustment of cell shape and adhesion, via the contraction of basal keratinocyte filopodia in the follicle, may contribute to the regulation of branching morphogenesis of pennaceous feathers [28]. Here, we investigate the embryonic development of the plumulaceous down-type feathers in the chicken.

The sonic hedgehog (Shh) pathway is a key regulator of diverse developmental processes [29,30]. Canonical Shh signaling begins with the SHH ligand binding to its receptor Patched (PTCH), thereby reversing the repression of the transmembrane protein Smoothened (SMO). The latter subsequently activates signaling *via* the transcription factor GLI, an intracellular zinc finger protein that triggers downstream transcription [31–33]. The Shh pathway plays multiple roles during feather development [34]. First, we have shown that transient in vivo agonism of Shh pathway signaling in chicken at embryonic day 11 (E11), through a single intravenous injection of smoothened agonist (SAG), triggers a complete and permanent transition from reticulate foot scales to feathers [35]. Remarkably, the resulting juvenile down-type ectopic feathers subsequently transition into adult regenerative bilaterally symmetric contour feathers, without the need for sustained SAG treatment, indicating that the over-expression of the Shh pathway at E11 induces a permanent shift in developmental fate of the corresponding placodes. Hence, the Shh pathway is clearly involved in the specification of avian skin appendages. Second, *Shh* mediates feather bud outgrowth by regulating interactions between the epidermis and mesenchyme [36] and may mediate the proliferation of dermal progenitor cells during morphogenesis [37]. Third, the relative level of *Shh* signaling within the posterior edge of the wing is involved in determining the location of specialized pennaceous flight feathers [38]. Fourth, feather branching morphogenesis in the embryonic chicken requires the spatial patterning of *Shh* through a reaction-diffusion mechanism that also involves the bone morphogenetic protein, *Bmp2* [7,8]. In this proposed activator-inhibitor model, diffusing *Shh* activates its own transcription, as well as the transcription of *Bmp2*, which then inhibits *Shh* [8]. The stationary striped pattern of expression that arises from this mechanism provides a molecular template defining the position of individual feather barbs, *i.e.*, *Shh* and *Bmp2* are observed in characteristic longitudinal expression domains defining the edges of individual barb ridges [7,8]. Although replication-competent avian retrovirus infection has previously been used to manipulate the expression domains of *Shh* and *Bmp2* that dictate feather barb formation [8], functional in vivo evidence regarding the roles of *Shh* during subsequent feather morphogenesis remains limited.

Here, we investigate feather development in the chicken. First, we present light sheet fluorescence microscopy (LSFM) imaging data regarding the normal patterning and morphogenesis of embryonic feathers. Next, we use precise intravenous *in-ovo* injections [35,39] of sonidegib to pharmacologically inhibit Shh pathway signaling during feather development at embryonic day 9 (E9), *i.e.*, at the placodal stage preceding feather-bud outgrowth on the wings. This treatment temporarily modifies *Shh* expression to produce striped domains, temporarily arrests morphogenesis, and results in unbranched and non-invaginated feather buds—akin to putative proto-feathers—in embryos until E14. Although feather morphogenesis partially recovers, hatched sonidegib-treated chickens exhibit naked regions of the skin surface with dormant and perturbed follicles. Remarkably, these dormant follicles are subsequently reactivated before seven weeks of post-embryonic development. Using bulk RNA sequencing (RNAseq) and a rescue experiment (with SAG), we show that sonidegib specifically reduces Shh pathway signaling. Overall, we provide comprehensive functional evidence for the role of the Shh pathway in mediating feather morphogenesis in the chicken, supporting the hypothesis that modified Shh signaling has contributed to the evolutionary diversification of feathers.

## Results

### Feather morphogenesis in the chicken embryo

The feathers of hatched chickens can be categorized into two groups, including simple plumulaceous feathers (i.e., down-type feathers) that lack a central ridge known as a rachis, and the more ordered pennaceous feathers that do exhibit a rachis (i.e., flight and contour feathers) (Fig 1A). Using volumetric LSFM, we describe the development of feathers in the embryonic chicken from E9 to E14 (Fig 1B and 1C). By E9, feather placodes cover the chicken’s dorsum and exhibit local expression of *Shh* (Fig 1B, left panel) [21]. By E10, we observe significant feather bud outgrowth across the body (Fig 1B, right panel), with *Shh* expression becoming localized to the distal tips of developing units [9,37]. Feather buds subsequently propagate across the wings of the embryonic chicken (Fig 1C). At E10, the first placodes can be seen on the posterior edge of the wing. By E11, feather bud coverage across the wings is complete, with a developmental gradient observable from posterior, with the most advanced buds, to anterior, with the least advanced buds. Feather bud outgrowth continues throughout E12, at which stage the longitudinal domains of increased cell density defining the position of future barb ridges become visible. Additionally, optical LSFM sections reveal epidermal SHH immunofluorescence in feather buds on the wing from E10 to E12. Keratinization of feather buds restricts the use of SHH antibodies beyond E12. Invagination of wing feather buds and formation of the follicle are observable from E13 onwards. Overall, feather morphogenesis requires outgrowth, branching, and invagination, and previous research has demonstrated that the Shh pathway is a key regulator of each of these three developmental processes [7–9,34,37].

### Sonidegib treatment temporarily changes Shh expression pattern and arrests feather morphogenesis

To experimentally investigate the role of the Shh pathway in mediating feather bud morphogenesis, we employ our recently published protocol for the rapid intravenous injection of developing amniote embryos [16,35,39]. This technique facilitates drug delivery directly into the vascular system of embryos developing within a hard-shelled egg. To precisely antagonize the Shh pathway, we injected chicken embryos with sonidegib (also called NVP-LDE225), a specific and potent inhibitor of Smoothened (Smo) [32,40]. Embryos were treated with a single injection of sonidegib at E9 to perturb feather bud development on the body and wings (Fig 1B and 1C), before fixation and analysis at subsequent embryonic and post-embryonic stages (see Materials and methods for further details).

Control samples treated with DMSO exhibit normal outgrowth of feather buds across the body and the wings from E10 to E14, producing elongated and filamentous feather buds (S1A, S2–S6 Figs). Conversely, sonidegib-treated embryos exhibit a dose-dependent reduction in feather bud outgrowth (S1B–S1D, S2–S6 Figs), with higher sonidegib doses resulting in increasingly restricted outgrowth. Indeed, samples treated with 300 µg sonidegib (the highest dose) exhibit small and nodular feather buds at E14 (S1D, S2–S6 Figs).

**Fig 2:**
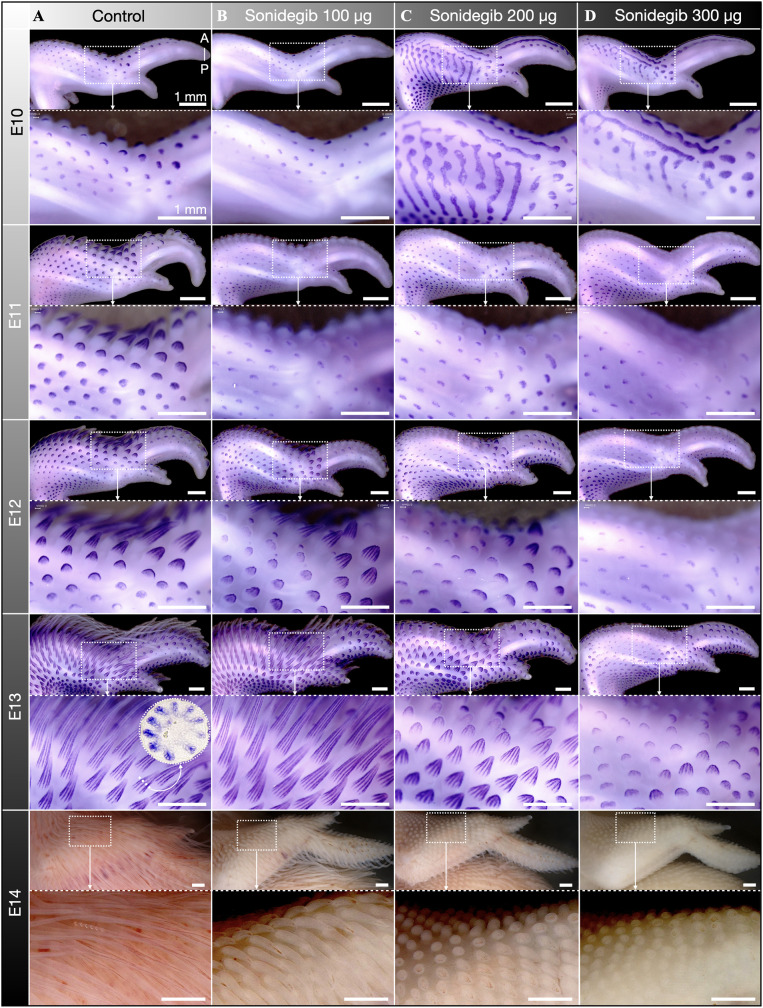
WMISH reveals that sonidegib treatment arrests feather branching morphogenesis. (**A**) The feathers of DMSO-treated control samples undergo normal patterning and morphogenesis [9], including the emergence of longitudinal *Shh* expression domains associated with branching morphogenesis from E12 to E13 [7,8]. (**B**) Samples treated with 100 µg sonidegib exhibit similar feather patterning to control samples but with slightly perturbed feather bud outgrowth (units are shorter across comparable embryonic stages). (**C**, **D**) Samples treated with either 200 or 300 µg sonidegib exhibit large striped expression domains of *Shh* at E10, demonstrating that these treatments temporarily modify the underlying reaction-diffusion dynamics. Normal spotted patterning recovers by E11 with *Shh* expression restricted to individual feather domains. However, subsequent morphogenesis from E11 to E13 remains perturbed as feather buds undergo substantially less outgrowth than in control samples. (**D**) At E13, samples treated with 300 µg sonidegib also reveal a reduction in longitudinal *Shh* expression domains associated with feather branching morphogenesis. (**A–D**, bottom row) As *Shh* WMISH becomes problematic after E13, only the morphological effect of sonidegib-induced dose-dependent reduction in feather bud outgrowth is shown at E14 (also shown in S1 Fig).

To investigate how our sonidegib treatment impacts the local gene expression of *Shh* during feather bud development on the wings, we use whole mount in situ hybridization (WMISH) (Fig 2). In control samples at E10, we observe the local expression of *Shh* in immature feather buds partially covering the wings (Fig 2A). By E11, *Shh* expression becomes focused in the distal tip of outgrowing units [9,37]. From E12 to E13, feather buds become dramatically elongated whilst *Shh* becomes restricted to the characteristic longitudinal expression domains that determine the formation of individual feather barbs [7,8]. In samples treated with 100 µg sonidegib, spatial expression patterns of *Shh* remain similar to those observed in control samples (Fig 2B). However, feather bud outgrowth becomes restricted, with units appearing slightly smaller across comparable embryonic stages. Remarkably, samples treated with either 200 or 300 µg sonidegib exhibit dramatically modified *Shh* expression patterns at E10, with large striped domains of *Shh* appearing instead of the characteristic spots associated with individual feather placodes (Fig 2C and 2D). Note that similar results have been reported ex vivo with feather-forming tissue explants treated with cyclopamine, a drug with multiple effects, including antagonism of the Shh pathway [37]. Our results indicate that a reduction of Shh signaling, an activator of early feather patterning [9,18], modifies the underlying reaction-diffusion dynamics to produce a pattern of stripes instead of spots. Remarkably, by E11 *Shh* expression recovers its spotted pattern associated with individual feather domains (Fig 2C and 2D), demonstrating that reaction-diffusion patterning robustly resists temporary pharmacological down-regulation of Shh signaling. However, although feather bud patterning recovers by E11, subsequent morphogenesis remains perturbed: from E12 to E13 these feather buds undergo substantially less outgrowth (Fig 2C and 2D) than observed in control samples. At E13, in samples treated with 300 µg sonidegib, this results in a dramatic reduction in the longitudinal *Shh* expression domains associated with branching morphogenesis (Fig 2D). At E14, as keratinization of feathers restricts the use of WMISH, we instead show the morphological impact of sonidegib treatment on the wings (Fig 2B–2D, bottom row). Again, these data reveal a dose-dependent reduction in feather bud outgrowth as samples treated with 300 µg sonidegib exhibit greatly shortened and unbranched feather buds.

**Fig 3:**
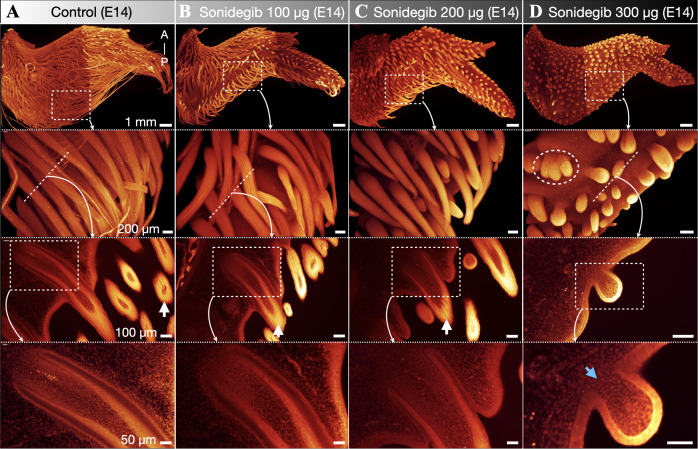
Sonidegib treatment arrests feather bud outgrowth and invagination. We use LSFM of nuclear stained (TO-PRO-3) chicken wings at E14 to investigate the morphological effect of sonidegib treatment. (**A**) Control samples exhibit normal development of elongated feather buds with well-advanced follicles and visible barb ridges (white arrow). (**B**, **C**) We observe a dose-dependent effect of sonidegib treatment, with higher doses resulting in shorter feather buds and less advanced follicle development. (**D**) Samples treated with 300 µg sonidegib exhibit minimal feather bud outgrowth, the fusion of feather buds (white oval dotted outline), absent branching morphogenesis, and no follicle invagination (blue arrow).

Next, we use LSFM of nuclear-stained (TO-PRO-3) chicken wings to examine the morphological effect of sonidegib treatment at E14 (Fig 3). Again, we observe a dose-dependent effect of treatment upon feather bud outgrowth, with higher doses resulting in shorter feather buds (Fig 3A–3D). In addition to a dramatic reduction in outgrowth, we also observe substantial variation in the shapes and sizes of feather buds in samples treated with 300 µg sonidegib, including the fusion of individual units (Fig 3D, oval outline). Importantly, feather branching morphogenesis is arrested at E14 by sonidegib treatment. Indeed, although feather barbs are present in control and lower-dose treatments (Fig 3A–3C, third row of panels, white arrows), samples treated with 300 µg sonidegib reveal no branching at E14 (Fig 3D, two lowest panels). Hence, although some longitudinal *Shh* expression domains are visible at E13 (Fig 2D, lowest panel), this gene expression is not translated into a corresponding morphological pattern of cell densities by E14. Furthermore, LSFM reveals that the invagination (inward growth) of feather follicles is perturbed by sonidegib treatment, as samples treated with higher doses exhibit less advanced follicles (Fig 3A–3C). Remarkably, feather bud invagination is entirely absent in samples treated with 300 µg sonidegib (Fig 3D, two lowest panels, blue arrow). Therefore, at high doses, sonidegib treatment strongly perturbs feather bud morphogenesis, restricting feather outgrowth, follicle invagination and branching morphogenesis (Fig 3D).

To investigate whether the morphological effects of sonidegib treatment constitute permanent modifications to feather morphology, we next examined treated embryonic samples fixed at later developmental stages. By E17, we observe a partial recovery of feather morphogenesis, with sonidegib-treated embryos exhibiting long, filamentous, and keratinized feathers (S7 Fig). However, these feathers remain shorter in samples treated with higher sonidegib doses and their coverage remains sparse in embryos treated with 300 µg sonidegib. At E21, we observe this same dose-dependent effect of decreased feather elongation and coverage with increasing strengths of sonidegib treatment (S8 Fig). These results demonstrate that, although treatment with 300 µg sonidegib strongly perturbs feather morphogenesis until E14 (Figs 2–3), this developmental process subsequently undergoes a partial recovery, presumably as the inhibitory effect of sonidegib declines (S7 and S8 Figs).

**Fig 4:**
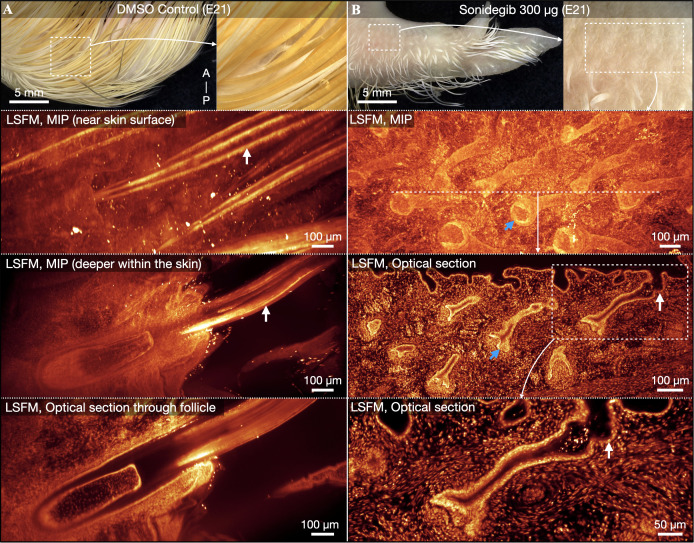
Naked skin regions in sonidegib-treated embryos contain perturbed follicles at E21. (**A**) Control samples exhibit normal coverage of keratinized and filamentous feathers. LSFM of nuclear stained (TO-PRO-3) control samples reveals deeply embedded feather follicles from which highly keratinized feather shafts emerge, consisting of multiple individual barbs (white arrows). (**B**) The anterior wings of samples treated with 300 µg sonidegib exhibit naked regions of skin adorned with perturbed sub-epidermal feather follicles (blue arrows) that completely lack the outgrowth of feather buds (white arrows), and exhibit less tissue differentiation than observed in control samples. Therefore, sonidegib treatment does not abolish the patterning of feather follicles, but instead dramatically perturbs feather bud and follicle morphogenesis in these regions, resulting in naked areas of the skin.

Samples treated with high doses of sonidegib also exhibit naked patches of skin at late embryonic stages (S7 and S8 Figs), including in the anterior region of the wing at E21 (Fig 4, top panels). We next investigate these samples using LSFM of nuclear staining (TO-PRO-3). The wings of E21 control samples exhibit normal fully developed, deeply embedded feather follicles, accompanied by highly keratinized and filamentous feather shafts (Fig 4A). Unexpectedly, naked skin regions of samples treated with 300 µg sonidegib do exhibit invaginated feather follicles; however, these structures are deformed and are not associated with feather outgrowths (Fig 4B). Furthermore, these follicles are substantially less developed than those of control samples and exhibit less differentiated and less keratinized tissues. Therefore, rather than abolishing or modifying the spatial patterning of feather follicles in naked regions of the skin at E21, treatment with 300 µg sonidegib at E9 both aborts feather bud outgrowth and partially reduces follicle development. However, these perturbed feather follicles remain present beneath the skin.

### Sonidegib treatment specifically down-regulates Shh pathway activity

**Fig 5:**
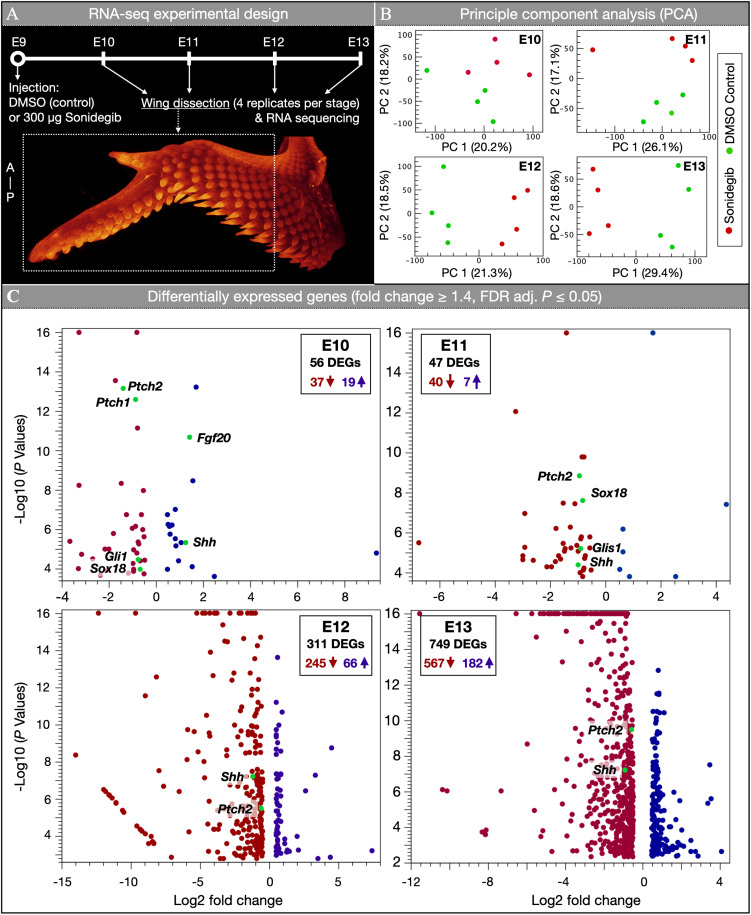
Bulk RNA sequencing reveals that sonidegib treatment down-regulates Shh pathway signaling. (**A**) Samples were injected with either DMSO (controls) or 300 µg sonidegib at E9. Wings were then dissected for RNA sequencing from E10 to E13, with four biological replicates used for each treatment at each stage. (**B**) From E11 onwards, PCA reveals a clear separation between control and treated samples. (**C**) Differentially expressed genes (DEGs) (filtered by a fold change of ≥1.4 and a false-discovery rate (FDR) adjusted *P*-value of ≤0.05) are shown in volcano plots for four embryonic stages. At E10, Shh pathway members *Ptch1*, *Ptch2* and *Gli1* are down-regulated. From E11 to E13, both *Ptch2* and *Shh* are persistently down-regulated in treated samples relative to controls. See file S1 Data for the data underlying the graphs shown in the figure.

We next sought to elucidate the molecular mechanisms underpinning the effect of sonidegib treatment using bulk RNA-seq analysis, to confirm the specific inhibitory effect of this drug on the Shh pathway [32,40]. Embryos were injected at E9 with either DMSO as a control or 300 µg sonidegib, and fixed daily at four subsequent developmental stages from E10 to E13. RNA was extracted for sequencing from dissected embryonic wing tissue from four biological replicates at each of the four developmental time points (Fig 5A) (see Materials and methods for details).

First, to assess the reliability of treatment across individual biological replicates, we use Principal Component Analysis of our RNA-seq dataset. This reveals a clear separation of sonidegib-treated (Fig 5B, red dots) versus DMSO control replicates (green dots) at each embryonic stage. Second, using a cluster analysis, we confirm that both sonidegib-treated and control samples are clearly distinguishable by their transcriptomic profiles (S9 Fig). Indeed, after filtering differentially expressed genes (DEGs) with a false-discovery rate (FDR) adjusted *P*-value of ≤0.05, the corresponding heat maps show that, at each time point, individual replicates cluster together according to their treatment type. Third, we identify key DEGs between sonidegib-treated and control samples (Fig 5C). To define DEGs, we filtered our dataset using a fold change of ≥1.4 and an FDR adjusted *P*-value of ≤0.05 (see Supporting information file S1 for both filtered and unfiltered DEGs).

At E10, i.e., 1 day after sonidegib treatment, we detect 56 DEGs (37 down-regulated and 19 up-regulated) and we already observe strong down-regulation of the SHH receptors *Ptch1* and *Ptch2*, as well as the downstream target *Gli1*. Note that we also observe a small increase in the expression of *Shh* itself, which may be indicative of an initial compensatory response following Shh pathway inhibition. We also observe strong up-regulation of fibroblast growth factor 20 (*Fgf20*), a key regulator of early feather bud development [26], which again may constitute a compensatory response. Our results also reveal weak down-regulation of SRY-related HMG-box 18 (*Sox18*), which has previously been observed in developing feather buds [41] and can induce the development of ectopic feathers when mis-expressed [42].

At E11, we detect 47 DEGs (40 down-regulated and 7 up-regulated). This includes the down-regulation of *Ptch2* and Gli-like transcription factor 1 (*Glis1*), demonstrating the continuation of decreased Shh pathway activity. Note that *Shh* itself is now also down-regulated, suggesting that the initial compensatory response to the treatment observed at E10 is now arrested. We also observe the down-regulation of *Sox18* at this stage. By E12, we identify 311 DEGs (245 down-regulated and 66 up-regulated), which again includes suppressed expression of both *Ptch2* and *Shh* itself. This sustained repression of *Shh* indicates that sonidegib treatment triggers negative feedback of intrinsic *Shh* signaling. At E13, we detect 749 DEGs (567 down-regulated and 182 up-regulated). Again, this includes the down-regulation of both *Ptch2* and *Shh*. Importantly, these strong and temporally consistent responses of both *Ptch2* and *Shh* conform to our previous RNA-seq analysis of chicken embryos treated with SAG [35], which has an inverse effect on Shh signaling [31]. Note that the much larger numbers of DEGs at E12 and E13 (in comparison to E10 and E11) are expected as the perturbation of Shh signaling impacts numerous downstream targets of the transcription factors included in the Shh pathway. Overall, our analysis reveals that Shh pathway members are consistently and negatively differentially expressed in response to sonidegib treatment at E9 (Fig 5C).

Next, we present the temporal expression dynamics for genes associated with skin appendage development in mean transcripts per million (TPM) (S10 Fig). These results reveal the down-regulation of Shh pathway members, including *Shh*, *Ptch1*, *Ptch2*, and *Gli1*, in response to sonidegib treatment (S10A Fig, top row of graphs). We also observe strong down-regulation of hedgehog interacting protein *Hhip* (S10A Fig, second graph in bottom row). In line with the above DEG analyses, our TPM analyses (S10 Fig) show (i) no significant differential expression of *Smo* itself in response to the pharmacological targeting of this receptor [35] and (ii) temporary up-regulation of *Fgf20* at E10, which may constitute a compensatory effect of Shh pathway inhibition. Note that the fibroblast growth factor binding protein 1 (*Fgfbp1*) and EDAR-Associated Via Death Domain (*Edaradd*) are both significantly down-regulated at E12 or E13 (S10B Fig), similar to *Sox21*, and wingless/integrated (Wnt) family members *Wnt6*, *Wnt16* and *Wnt10a* (S10C Fig). This is indicative of their roles in late feather morphogenesis, which are suppressed in sonidegib-treated samples (Figs 2 and 3). Finally, we observe a dramatic down-regulation of genes associated with Keratin (*Krt*) production, including *Krt14*, *Krt6a*, *Krt23*, *Krt40* and feather keratin 1-like (*Fk1-like*) (S10D Fig). Again, we attribute this to the arrested morphogenesis of feather buds in sonidegib-treated samples. Overall, these results confirm that Shh pathway activity is specifically down-regulated [32,40] in developing feather buds, in response to a single intravenous injection with sonidegib at E9.

To further investigate our transcriptomic data, we next present a gene ontology (GO) enrichment analysis of “biological processes” [43] associated with our DEG sets (fold change of ≥1.4 and FDR-adjusted *P* ≤ 0.05) from each time point. The 10 most significant GO terms are reported for each stage, along with the number and names of the detected DEGs (S11 Fig). At both E10 and E11, *Shh* is associated with all of the 10 most significant GO terms, including “regulation of cell communication” and “regulation of signaling” (S11A and S11B Fig). Shh pathway members, including *Ptch1*, *Ptch2*, *Gli1* and *Glis1*, also frequently contribute to significant GO terms throughout these stages. At E12, *Shh* is associated with nine of the 10 most significant GO terms, the most significant of which is “regulation of cell differentiation” (S11C Fig). At E13, we observe *Shh* in 5 of the 10 most significant GO terms, including “regulation of gene expression” (S11D Fig). Overall, our GO enrichment analysis reveals that sonidegib treatment is significantly associated with the regulation of Shh pathway-associated biological processes.

Finally, we present a Kyoto Encyclopedia of Genes and Genomes (KEGG) pathway analysis of DEG sets from each time point (S12A Fig) [44]. At E10, the most significant detected KEGG pathway is “Hedgehog signaling”, with numerous Shh pathway members differentially expressed, including *Shh*, *Ptch*, *Gli*, and *Hhip* (S12B Fig, DEGs shown in red). Across all subsequent stages, the most consistently detected significant KEGG pathway is “Basal cell carcinoma” which involves Shh pathway members, including *Shh* and *Ptch* (S12C–S12E Fig, DEGs shown in red). Overall, our analysis demonstrates that Shh-associated KEGG pathways are significantly differentially regulated following sonidegib treatment.

### Reactivation of the Shh pathway rescues feather morphogenesis

**Fig 6:**
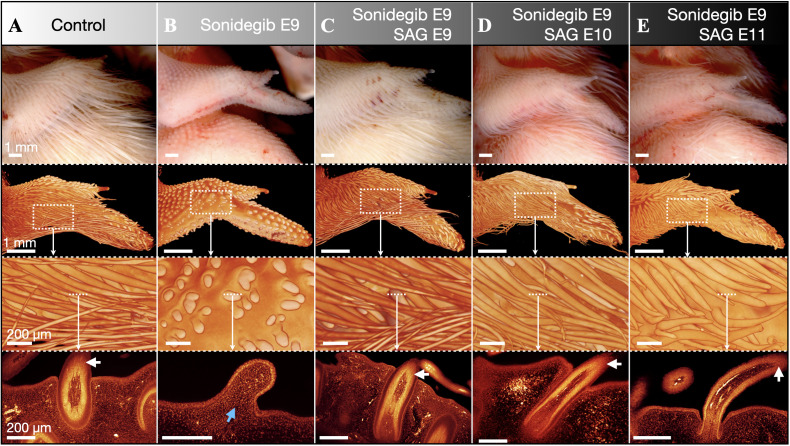
SAG treatment rescues sonidegib-perturbed feather morphogenesis at E14. Embryos were treated with SAG, a SMO agonist, together or following sonidegib delivery at E9. (**A**) Control embryos injected with DMSO at E9 exhibit normal elongated, filamentous feathers at E14. (**B**) Embryos treated at E9 with 300 µg sonidegib exhibit at E14 substantially reduced units that lack both branching and follicle invagination (blue arrow). (**C**) However, the combined injection of 300 µg sonidegib and 50 µg SAG at E9, prevents abnormal morphogenesis of feather buds. (**D**) Treatment with 100 µg SAG at E10 rescues feather morphogenesis in samples treated with 300 µg sonidegib at E9. (**E**) Treatment with 150 µg SAG at E11 only partially rescues feather morphogenesis in samples treated with 300 µg sonidegib at E9 (*i.e.*, feather buds remain shorter at E14) probably because of a shorter recovery time (*i.e.*, E11–E14). LSFM sections in the bottom panels reveal normal feather branching (white arrows) and follicle morphogenesis in the control and in all samples treated with both sonidegib and SAG, although samples treated with SAG at E11 exhibit reduced follicle invagination (E, bottom panel).

We next sought to investigate the specificity of the effect by which sonidegib perturbs embryonic feather development. Therefore, we repeated our sonidegib treatments in combination with the delivery of SAG (Figs 6 and S13). Contrary to sonidegib, which inhibits Shh pathway activity, SAG is a Shh pathway activator [35]. This rescue experiment allows us to test the specificity of sonidegib on SMO because both sonidegib and SAG are considered to act directly, albeit with opposing effects, on this key transducer of the Shh pathway [31,32]. Embryos were treated at different embryonic stages with SAG, together or following 300 µg sonidegib treatment at E9. The precise dosage of SAG was adjusted according to approximate embryo weight, with more developed embryos receiving larger doses. All embryos were fixed at E14 to examine the effects of these treatments on feather morphogenesis (see S13 Fig for all replicates).

Control embryos treated with DMSO at E9 develop normal filamentous feather buds by E14 (Fig 6A, see also Fig 3), whilst embryos treated with 300 µg sonidegib exhibit substantially reduced units that lack both branching and follicle invagination (Fig 6B). However, samples treated with a combined injection of 300 µg sonidegib and 50 µg SAG at E9 display normal elongated feather buds (Fig 6C). Samples treated with 300 µg sonidegib at E9 followed by 100 µg SAG at E10 exhibit similarly elongated feather buds (Fig 6D). When the two injections are separated by a longer interval (300 µg sonidegib at E9 followed by 150 µg SAG at E11) the treated embryos exhibit a less complete rescue of the feather phenotype (i.e., with less elongated feather buds; Fig 6E). This latter result could be due to a reduced receptivity to SAG at E11 or, more simply, to the reduced recovery time between SAG treatment at E11 and fixation at E14. LSFM sections reveal that all samples treated with both sonidegib and SAG undergo normal feather branching and follicle invagination (Fig 6C–6E, bottom row), although follicle development is slightly less advanced in samples treated with sonidegib at E9 and SAG at E11 (Fig 6E, bottom panel). Overall, these experiments demonstrate that the intravenous delivery of SAG is sufficient to robustly rescue feather morphogenesis following sonidegib treatment. Hence, this recovery of feather morphogenesis highlights the specificity of both drugs on the Shh pathway.

### Feather morphogenesis robustly recovers post-hatching

We next sought to uncover the effect of sonidegib treatment on feather development in post-embryonic chickens. Therefore, we allowed chickens treated with sonidegib at E9 to hatch and monitored their development until 49 days post-hatching (dph).

**Fig 7:**
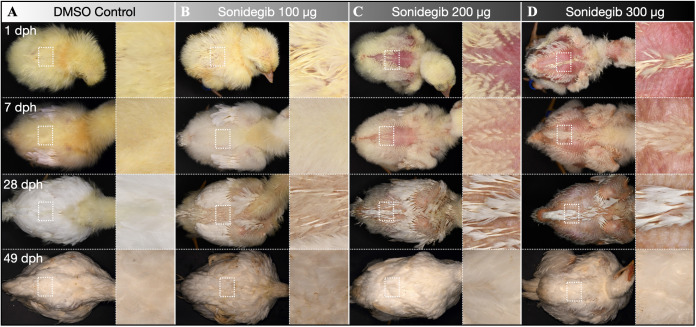
Post-embryonic effect of sonidegib treatment. (**A**) Control samples exhibit complete coverage of (yellow) plumulaceous down-type feathers from 1 dph (top row) until 7 dph (second row). By 28 dph (third row), down-type feathers are observed transitioning into (white) pennaceous contour feathers. This process is complete by 49 dph (bottom row). (**B**) Samples treated with 100 µg sonidegib exhibit slightly more sparsely patterned feathers, with some patches of skin visible through their plumage. However, by 49 dph, their feather coverage appears indistinguishable from controls. (**C**, **D**) Samples treated with either 200 or 300 µg sonidegib exhibit large regions of naked skin on their backs, from 1 dph to 28 dph. Upon closer inspection, these naked regions exhibit regularly patterned skin elevations, which likely correspond to dormant subepidermal follicles (as shown in Fig 4B). By 49 dph, the feather coverage of samples treated with 200 or 300 µg sonidegib recovers and appears comparable to both controls and samples treated with 100 µg sonidegib.

At 1 dph, we observe a dose-dependent effect of sonidegib treatment (Fig 7, first row). Control chickens exhibit normal coverage of feathers (Fig 7A). However, feather coverage in samples treated with 100 µg sonidegib appears more sparse, with some patches of skin visible through the plumage (Fig 7B). Samples treated with either 200 or 300 µg sonidegib exhibit large areas of naked skin, particularly on the back (Fig 7C–7D). Upon closer inspection of these naked regions, we observe regularly patterned elevations of the skin (Fig 7D, top-right panel). As observed on naked regions of the wing at E21 (Fig 4B, top-right panel), these low protrusions likely correspond to dormant feather follicles. Next, we removed feathers from the dorsal midline to compare their morphologies (S14 Fig). Interestingly, we observe a dose-dependent treatment effect, with higher sonidegib doses resulting in smaller down-type feathers. All these results indicate that Shh pathway antagonism at E9 perturbs feather morphogenesis in newly hatched chickens.

At 7 dph, we observe this same dose-dependent effect of sonidegib treatment on feather coverage (Fig 7, second row). By 28 dph, we observe the widespread transition from down-type feathers into pennaceous adult contour feathers in all hatched chickens (Fig 7, third row), and naked skin regions persist on the lateral back surface of samples treated with higher doses of sonidegib (Fig 7C–7D, third row). However, by 49 dph, these naked regions are no longer visible, and the feather coverage of different treatments is now difficult to distinguish (Fig 7, fourth row). By removing the feathers from these samples at 49 dph, we reveal the patterning of feather follicles across the backs and wings of each treatment group (S15 Fig). Follicle patterning appears comparable between control chickens and those injected with different doses of sonidegib. Therefore, although sonidegib treatment at E9 temporarily arrests feather morphogenesis at earlier stages, it does not perturb the spatial distribution of follicles at 49 dph.

Overall, our results reveal that Shh pathway antagonism via intravenous sonidegib injection gives rise to dormant feather buds at the hatching stage, corresponding to naked regions of the skin (Fig 7). These naked regions persist until 28 dph. However, dormant follicles are subsequently re-activated by 49 dph, perhaps during the first natural molting cycle of the chicken, resulting in normal feather coverage.

Previous research has shown that the relative level of Shh signaling within the posterior edge of the wing is involved in determining the location of specialized flight feathers [38]. Flight feathers are large pennaceous feathers that emerge in precisely patterned rows from the posterior region of either wing (Fig 1A). In accordance with previous work, our sonidegib treatments also perturb flight feather specification (S16 Fig) [38]. Flight feathers are clearly visible on the wings of control samples at 7 dph (S16A Fig, second row). However, these units become smaller in samples treated with increasing doses of sonidegib and are almost entirely absent in birds treated with the highest dose (S16D Fig). This effect persists at both 28 dph (S16 Fig, third row) and 49 dph (S16 Fig, fourth row), with smaller contour-type feathers instead adorning the wing. Therefore, this modification to flight feather identity constitutes a permanent modification to the patterning of the integument.

## Discussion

Overall, our results demonstrate that transient Shh pathway antagonism at E9 temporarily arrests feather morphogenesis in the chicken. Treatment with 300 µg sonidegib halts both feather bud outgrowth and follicle invagination until E14. Although morphogenesis subsequently recovers in some regions (S7 and S8 Figs), newly hatched chickens exhibit a dose-dependent reduction in feather size along the dorsal midline (S14 Fig). Naked skin regions associated with dormant feather follicles are observed both at E21 (Fig 4B) and after hatching, until 28 dph (Fig 7). These dormant follicles are subsequently reactivated, perhaps during the first natural molting cycle of the chicken, giving rise to normal feather coverage by 49 dph. Therefore, Shh pathway antagonism at E9 temporarily restricts feather emergence in embryonic and hatched chickens but does not modify the final adult spatial patterning of feather follicles.

Previous research has shown that Shh pathway inhibition can perturb chicken feather bud patterning and outgrowth in an ex vivo tissue culture system [37]. Here, we demonstrate that such an effect of Shh pathway inhibition is temporary when examined in vivo. Initially (i.e., one day after sonidegib treatment), we observe the dramatically modified spatial expression of *Shh* (Fig 2C–2D), which forms a pattern of broad stripes instead of the characteristic spots associated with individual feather buds. This indicates that the reaction-diffusion dynamics underlying feather patterning are initially strongly modified by Shh pathway inhibition. Although the normal spotted pattern recovers by E11, this treatment strongly affects subsequent feather morphogenesis at E14 (Figs 2 and Fig 3) and also at later embryonic (S7 and S8 Figs) and post-hatching stages (Fig 7). However, at 49 dph, the development of feathers recovers, with the notable exception of the specialized flight feathers. Overall, our results demonstrate that both the in vivo patterning and morphogenetic processes underlying feather development robustly resist pharmacological down-regulation of Shh pathway signaling.

These results strongly contrast with our previous converse experiment of transiently up-regulating Shh pathway signaling at E11 in the embryonic chicken, which results in the permanent transformation of reticulate scales into feathers [35]. Importantly, the timing of this up-regulation coincides with the emergence of reticulate scale signaling placodes on the ventral footpad. Here, our Shh pathway inhibition at E9 also coincides with the emergence of feather placodes across the wings (Fig 1). However, the effects of sonidegib treatment are temporary, with perturbed feathers subsequently recovering at later stages. Identifying the mechanisms underlying this distinction in the developmental plasticity of feathers *versus* reticulate scales would require the full characterization of the two corresponding gene interaction networks involved in their development.

The ability of feather follicles to eventually fully recover at 49 dph may be due to the presence of a dermal population of stem cells in the feather follicle, i.e., the persistence of these cells may facilitate the recovery of feather growth following pharmacological developmental perturbations. Conversely, avian scales (including reticulate scales) are not cyclically replaced and are associated with neither a follicle nor a dermal population of stem cells, explaining the capacity to induce a full and permanent transition from reticulate scales to feathers through Shh pathway agonism at E11 [35]. Furthermore, we suggest that flight feathers do not recover in individuals treated with the highest dose (300 µg) of sonidegib because the transient increase in Shh signaling required for their specification is perturbed at the appropriate stage of embryonic development [38].

Feather branching morphogenesis has been attributed to a molecular activator-inhibitor mechanism, involving *Shh* and *Bmp2*, that defines the spatial distribution of individual barb ridges [7,8]. It has been suggested that the establishment of this mechanism gave rise to the evolution of plumulaceous down-type feathers with multiple barb ridges [8], from a simple ancestral monofilamentous feather type [4]. Our in vivo experiments reveal that Shh pathway inhibition temporarily arrests feather bud branching, resulting in small and nodular feather buds at E14 (Fig 3D). These results support the hypothesis that modified Shh pathway signaling has contributed to (i) the evolutionary transition from proto-feathers to more elaborated *bona-fide* feathers and (ii) the diversification of feather morphologies across species.

Sonidegib is a selective and potent Shh pathway inhibitor that interacts with SMO to prevent downstream signaling [32,40]. The oral delivery of sonidegib has been used as a pharmacological treatment for advanced basal cell carcinoma, because aberrant hedgehog pathway activation is a major cause of this cancer type [45,46]. Interestingly, our KEGG pathway analysis identifies that, for all time points, sonidegib treatment affects the regulation of the “basal cell carcinoma” pathway (which involves the differential expression of both *Shh* and *Ptch*; S12 Fig). Although the half-life of sonidegib following oral delivery in humans is known to be 28 days, with a peak concentration at 2–4 h after ingestion [46], our data lacks the required temporal resolution to determine peak activity in the embryonic chicken. Furthermore, our results show that sonidegib treatment does not induce significant differential expression of *Smo* itself (S10A Fig). This is consistent with our previous study in which we activated Shh pathway signaling via smoothened using SAG, which did not result in significant differential expression of *Smo*, although *Ptch2* and *Shh* were both strongly upregulated [35]. Finally, by treating embryos with SAG following sonidegib delivery, we successfully induce the robust recovery of feather bud outgrowth, branching and follicle invagination (Fig 6). Hence, this recovery supports that SAG acts directly and inversely on the same target (SMO) as sonidegib [31,32].

In conclusion, we show that transient antagonism of the Shh pathway strongly perturbs feather development, by restricting feather bud outgrowth, invagination and branching morphogenesis. However, this effect is temporary, as feather development exhibits a near-complete post-embryonic recovery by 49 dph. Overall, this highlights the robustness of feather patterning as a developmental process. Finally, our study demonstrates the importance of in vivo experiments for obtaining a comprehensive understanding of developmental systems.

## Materials and methods

### Animal husbandry

Broiler chicken (Ross) eggs were obtained from a commercial chicken farm (La Prairie, 1721 Cournillens, Switzerland), and incubated at 37.5 °C with ~40% relative humidity. Maintenance of, and experiments with, all chicken embryos were approved by the Geneva Canton ethical regulation authority (authorization GE10619B) and performed according to Swiss law. These guidelines meet the international standards. All non-fluorescent imaging of chicken embryos was undertaken with a Keyence VHX 7000 digital microscope or a Nikon D800 camera.

### Light sheet fluorescence microscopy (LSFM)

LSFM was undertaken as previously described [35]. In brief, chicken samples stored in methanol (MeOH) were rehydrated and permeabilised in phosphate-buffered saline with gelatin, sodium azide, saponin, and Triton X-100. Samples were incubated with an Alexa Fluor-conjugated SHH antibody (sc3655112, Santa Cruz Biotechnology), before nuclear staining with TO-PRO-3 Iodide (Thermo Fisher Scientific). Tissue clearing was undertaken according to the iDISCO+ protocol [47]. Imaging was undertaken using the Ultramicroscope Blaze (Miltenyi Biotec). Volumetric data was processed and visualized using Imaris (Oxford Instruments, UK).

### In-ovo sonidegib treatment

Intravenous injections of chicken eggs were undertaken in accordance with our previously published work [16,35,39]. Chicken eggs were incubated until E9 and cleaned with 70% ethanol. Suitable veins for injection were identified via candling, and a detailing saw (Micromot 50/E, Proxxon) was used to remove the eggshell while keeping the underlying membrane intact. Mineral oil was then applied to the exposed membrane to increase its transparency. Samples were then treated with a single intravenous injection of either 10 µl of DMSO (as a control) or 10 µl of DMSO containing sonidegib (S2151, Selleckchem), a selective and potent Shh pathway inhibitor [32,40]. We selected sonidegib as a pharmacological *Shh* antagonist instead of cyclopamine (a more classical Shh pathway inhibitor), due to the greater specificity of the former [32]. Details of replicates for sonidegib injections are shown in Table 1. Injections were undertaken with a Hamilton syringe attached to a micromanipulator (MM33 right, Marzhauser). For combined sonidegib and SAG experiments, embryos were treated with either a mixed injection of 300 µg sonidegib and SAG at E9, or an injection with 300 µg of sonidegib at E9 followed by a subsequent SAG injection at E10 or E11. The dosage of SAG was adjusted according to the approximate weight of the embryo (60 µg at E9, 120 µg at E10 or 180 µg at E11) [35]. Details of replicates for combined sonidegib and SAG injections are shown in Table 2. Following injection, eggs were cleaned with 70% ethanol, the eggshell window was covered with adhesive tape, and the eggs were returned to the incubator until the desired stage. All replicates from these experiments are shown in supporting information figures and summarized in Table 1.

**Table 1:** Summary of sonidegib treatment replicates.

Treatment group	Survival at stage of fixation (embryonic days)							
	E10	E11	E12	E13	E14	E17	Hatch	Total
Control	5/8 (63%)	5/8 (63%)	5/9 (56%)	5/7 (71%)	5/8 (63%)	5/8 (63%)	4/7 (57%)	34/55 (62%)
Sonidegib 100 µg	5/10 (50%)	5/12 (42%)	5/11 (45%)	5/10 (50%)	5/13 (38%)	5/10 (50%)	4/10 (40%)	34/76 (45%)
Sonidegib 200 µg	5/14 (36%)	5/12 (42%)	5/12 (42%)	5/15 (33%)	5/13 (38%)	3/10 (30%)	3/10 (30%)	31/86 (36%)
Sonidegib 300 µg	5/15 (33%)	5/14 (36%)	5/12 (42%)	5/15 (33%)	5/17 (29%)	3/10 (30%)	2/10 (20%)	30/93 (32%)

**Table 2:** Summary of combined sonidegib and SAG treatment replicates.

Survival at stage of fixation (embryonic days)	Treatment group				
	Control	Sonidegib E9	Sonidegib E9 and SAG E9	Sonidegib E9 and SAG E10	Sonidegib E9 and SAG E11
Survival at E14	7/12 (58%)	8/30 (27%)	8/35 (23%)	8/52 (15%)	8/44 (18%)

### Whole mount in situ hybridization (WMISH)

WMISH of embryonic chicken samples was undertaken as previously described [35]. Following WMISH, samples were post-fixed and imaged using a Keyence VHX 7000 digital microscope.

### RNA-sequencing (RNA-seq) experiments and analysis

Following treatment with either sonidegib or DMSO, entire wings were dissected from embryos at E10, E11, E12 and E13, placed into TRIzol (Sigma-Aldrich) and stored at −80 °C. Four biological replicates were acquired for each treatment at each stage. RNA was extracted with the Direct-zol RNA Miniprep Kit (Zymo Research). RNA-seq (Illumina TruSeq stranded mRNA- NovaSeq 6000 100 PE [paired-end] with 40M reads) was undertaken by Macrogen Europe (Amsterdam, the Netherlands). Reads were aligned to the *Gallus gallus* genome (bGalGal1.mat.broiler.GRCg7b) and analyzed using CLC Genomics Workbench (QIAGEN). KEGG pathway analysis [48] shown in S12 Fig was undertaken using ShinyGO [49] and visualized with Pathview [50]. All RNA-seq datasets analyzed in this study are provided in supporting information file S1, and raw RNA-seq data are provided at https://doi.org/10.5061/dryad.8pk0p2nzj.

## Supporting information

S1 Fig Sonidegib treatment arrests feather morphogenesis. Samples fixed following treatment with sonidegib at E9 were fixed from E10 to E14. (**A**) DMSO control samples reveal normal outgrowth of feather buds. (**B**–**D**) Sonidegib-treated samples exhibit a dose-dependent effect, with stronger sonidegib doses corresponding to less developed feather buds by E14.
(PDF)

S2 Fig Experimental replicates from sonidegib treatments at E10. Chicken embryos injected at E9 with either DMSO (controls) or sonidegib (100, 200, or 300 μg) are shown.
(PDF)

S3 Fig Experimental replicates from sonidegib treatments at E11. Chicken embryos injected at E9 with either DMSO (controls) or sonidegib (100, 200, or 300 μg) are shown.
(PDF)

S4 Fig Experimental replicates from sonidegib treatments at E12. Chicken embryos injected at E9 with either DMSO (controls) or sonidegib (100, 200, or 300 μg) are shown.
(PDF)

S5 Fig Experimental replicates from sonidegib treatments at E13. Chicken embryos injected at E9 with either DMSO (controls) or sonidegib (100, 200, or 300 μg) are shown.
(PDF)

S6 Fig Experimental replicates from sonidegib treatments at E14. Chicken embryos injected at E9 with either DMSO (controls) or sonidegib (100, 200, or 300 μg) are shown.
(PDF)

S7 Fig Experimental replicates from sonidegib treatments at E17. Chicken embryos injected at E9 with either DMSO (controls) or sonidegib (100, 200, or 300 μg) are shown. Blue arrows show naked regions of the skin.
(PDF)

S8 Fig Experimental replicates from sonidegib treatments at E21. Chicken embryos injected at E9 with either DMSO (controls) or sonidegib (100, 200, or 300 μg) are shown.
(PDF)

S9 Fig RNA-seq sample clustering at different developmental time points. The clustering of individual RNA-seq sample replicates was undertaken for all sonidegib-treated and control samples at four developmental stages, using Euclidean distance and complete linkage of clusters. After filtering differentially expressed genes (DEGs) with a false-discovery rate (FDR) adjusted *P*-value of ≤0.05, the corresponding heat maps show that, at each time point, individual replicates cluster together according to their treatment type. See file S1 Data for the data underlying the graphs shown in the figure.
(PDF)

S10 Fig Temporal changes in gene expression (RNA-seq. data; transcripts per million [TPM]) after sonidegib treatment. Individual genes are only shown if they exhibit differential expression (false-discovery rate adjusted *P*-value of ≤0.05) at a minimum of one embryonic stage, with the exceptions of Gli2 and Smo. Significant differential expression is shown with a yellow outline. (**A**) Multiple members of the Shh pathway are continuously down-regulated. Shh itself is down-regulated from E11 onwards. (**B**, **C**) Other skin appendage-associated genes are down-regulated at later developmental stages, indicative of their role in feather morphogenesis which is perturbed in treated samples. (**D**) We also observe dramatic down-regulation in genes associated with keratin production due to the absence of advanced feather bud morphogenesis in sonidegib-treated samples. See file S1 Data for the data underlying the graphs shown in the figure.
(PDF)

S11 Fig GO term enrichment analyses of differentially expressed gene sets at four developmental time points. The 10 most significant GO terms (within “biological processes”) are reported for DEG sets comparing sonidegib-treated and control samples at each developmental stage. (**A**, **B**) Shh is associated with the 10 most significant GO terms at E10 and E11. Other Shh pathway members—including Ptch1, Ptch2, Gli1 and Glis1—are also frequently reported. (**C**) At E12, Shh is reported in 9 of the 10 most significant GO terms, and (**D**) at E13, Shh is reported in five of the 10 most significant GO terms.
(PDF)

S12 Fig Most significant KEGG pathways detected from DEG sets at four developmental time points. KEGG pathway analysis of DEG sets comparing sonidegib-treated and control samples was used to identify key pathways at each time point. (**A**) The most significant detected KEGG pathways for each stage are listed. (**B**) At E10, “Hedgehog signaling” is the most significant KEGG pathway (individual DEGs present in our dataset are shown in red). (**B**–**E**) From E10 to E13, the most consistently detected significant KEGG pathway is ‘Basal cell carcinoma’, which is mediated by interactions of both Shh and Ptch.
(PDF)

S13 Fig Experimental replicates from combined sonidegib and SAG treatments. Chicken embryos were treated with either (**A**) DMSO at E9 as a control, or (**B**) 300 μg sonidegib at E9, or (**C**) both 300 μg sonidegib at E9 and 50 μg SAG at E9, or (**D**) both 300 μg sonidegib at E9 and 100 μg SAG at E10, or (**E**) both 300 μg sonidegib at E9 and 150 μg SAG at E11. All samples were fixed and imaged at E14.
(PDF)

S14 Fig Down-type feather morphology of hatched sonidegib-treated chickens at 1 dph. Removing down-type feathers from the dorsal midline of control and sonidegib-treated chickens reveals a dose-dependent decrease in feather size resulting from treatments of increasing strength.
(PDF)

S15 Fig Follicle patterning on the bodies and wings of sonidegib-treated chickens at 49 dph. Plucking the feathers from the bodies and wings of chickens injected at E9 with either DMSO (controls) or sonidegib (100, 200, or 300 μg) reveals no notable difference in the spatial distribution of feather follicles.
(PDF)

S16 Fig The development of flight feathers is permanently reduced in sonidegib-treated chickens. The wings of chickens injected at E9 with either DMSO (controls) or sonidegib (100, 200, or 300 μg) are shown from 1 dph to 49 dph. (**A**, **B**) Flight feathers can be observed emerging in both control and 100 μg sonidegib-treated samples at 1 dph (top row). These pennaceous feathers continue to develop until E49 (bottom row), at which stage they appear slightly smaller in samples treated with 100 μg sonidegib. (**C**, **D**) At 1 dph flight feathers are not visible on the wings of samples treated with 200 or 300 μg sonidegib. By 7 dph, some small and perturbed flight feathers emerge. However, by 49 dph, flight feathers are largely absent from both of these treatment groups, and the posterior region of the wing is instead covered with smaller contour feathers.
(PDF)

S1 Data Aligned RNA-seq expression data and differential gene expression analyses for each developmental stage are provided in supporting information file S1 Data. The entire raw RNA-seq dataset is provided at https://doi.org/10.5061/dryad.8pk0p2nzj.
(ZIP)

## Abbreviations:

DEGs: differentially expressed genes
*Edaradd*: EDAR-Associated Via Death Domain
E9: embryonic day 9
E11: embryonic day 11
FDR: false-discovery rate
FGF: fibroblast growth factor
*Fgf20*: fibroblast growth factor 20
*Fgfbp1*: fibroblast growth factor binding protein 1
Gli1: Glioma-associated oncogene homolog 1
*Glis1*: Gli-like transcription factor 1
GO: gene ontology
*Krt*: Keratin
KEGG: Kyoto Encyclopedia of Genes and Genomes
LSFM: light sheet fluorescence microscopy
PTCH: Patched
RNAseq: RNA sequencing
SAG: smoothened agonist
Shh: sonic hedgehog
SMO: Smoothened
*Sox18*: SRY-related HMG-box 18
TPM: transcripts per million
WMISH: whole mount in situ hybridization
Wnt: wingless/integrated

## References

[1] Prum RO. Development and evolutionary origin of feathers. J Exp Zool. 1999;285(4):291–306. doi: 10.1002/(sici)1097-010x(19991215)285:4<291::aid-jez1>3.3.co;2-0 10578107

[2] Prum RO, Brush AH. The evolutionary origin and diversification of feathers. Q Rev Biol. 2002;77(3):261–95. doi: 10.1086/341993 12365352

[3] Godefroit P, Sinitsa SM, Dhouailly D, Bolotsky YL, Sizov AV, McNamara ME, et al. Dinosaur evolution. A Jurassic ornithischian dinosaur from Siberia with both feathers and scales. Science. 2014;345(6195):451–5. doi: 10.1126/science.1253351 25061209

[4] Benton MJ, Dhouailly D, Jiang B, McNamara M. The early origin of feathers. Trends Ecol Evol. 2019;34(9):856–69. doi: 10.1016/j.tree.2019.04.018 31164250

[5] Yang Z, Jiang B, McNamara ME, Kearns SL, Pittman M, Kaye TG, et al. Pterosaur integumentary structures with complex feather-like branching. Nat Ecol Evol. 2019;3(1):24–30. doi: 10.1038/s41559-018-0728-7 30568282

[6] Chen C-F, Foley J, Tang P-C, Li A, Jiang TX, Wu P, et al. Development, regeneration, and evolution of feathers. Annu Rev Anim Biosci. 2015;3:169–95. doi: 10.1146/annurev-animal-022513-114127 25387232 PMC5662002

[7] Harris MP, Fallon JF, Prum RO. Shh-Bmp2 signaling module and the evolutionary origin and diversification of feathers. J Exp Zool. 2002;294(2):160–76. doi: 10.1002/jez.10157 12210117

[8] Harris MP, Williamson S, Fallon JF, Meinhardt H, Prum RO. Molecular evidence for an activator-inhibitor mechanism in development of embryonic feather branching. Proc Natl Acad Sci U S A. 2005;102(33):11734–9. doi: 10.1073/pnas.0500781102 16087884 PMC1187956

[9] Jung HS, Francis-West PH, Widelitz RB, Jiang TX, Ting-Berreth S, Tickle C, et al. Local inhibitory action of BMPs and their relationships with activators in feather formation: implications for periodic patterning. Dev Biol. 1998;196(1):11–23. doi: 10.1006/dbio.1998.8850 9527877

[10] Sick S, Reinker S, Timmer J, Schlake T. WNT and DKK determine hair follicle spacing through a reaction-diffusion mechanism. Science. 2006;314(5804):1447–50. doi: 10.1126/science.1130088 17082421

[11] Harris MP, Rohner N, Schwarz H, Perathoner S, Konstantinidis P, Nüsslein-Volhard C. Zebrafish eda and edar mutants reveal conserved and ancestral roles of ectodysplasin signaling in vertebrates. PLoS Genet. 2008;4(10):e1000206. doi: 10.1371/journal.pgen.1000206 18833299 PMC2542418

[12] Di-Poï N, Milinkovitch MC. The anatomical placode in reptile scale morphogenesis indicates shared ancestry among skin appendages in amniotes. Sci Adv. 2016;2(6):e1600708. doi: 10.1126/sciadv.1600708 28439533 PMC5392058

[13] Cooper RL, Martin KJ, Rasch LJ, Fraser GJ. Developing an ancient epithelial appendage: FGF signalling regulates early tail denticle formation in sharks. Evodevo. 2017;8:8. doi: 10.1186/s13227-017-0071-0 28469835 PMC5414203

[14] Sengel. Morphogenesis of skin. New York: Cambridge University Press; 1976.

[15] Milinkovitch MC, Manukyan L, Debry A, Di-Poï N, Martin S, Singh D, et al. Crocodile head scales are not developmental units but emerge from physical cracking. Science. 2013;339(6115):78–81. doi: 10.1126/science.1226265 23196908

[16] Santos-Durán GN, Cooper RL, Jahanbakhsh E, Timin G, Milinkovitch MC. Self-organised patterning of crocodile head scales by compressive folding. Nature 2025; 637:375-383. 39663449 10.1038/s41586-024-08268-1 PMC11711089

[17] Turing AM. The chemical basis of morphogenesis. 1953. Bull Math Biol. 1990;52(1–2):153–97; discussion 119-52. doi: 10.1007/BF02459572 2185858

[18] Jiang TX, Jung HS, Widelitz RB, Chuong CM. Self-organization of periodic patterns by dissociated feather mesenchymal cells and the regulation of size, number and spacing of primordia. Development. 1999;126(22):4997–5009. doi: 10.1242/dev.126.22.4997 10529418

[19] Kondo S, Miura T. Reaction-diffusion model as a framework for understanding biological pattern formation. Science. 2010;329(5999):1616–20. doi: 10.1126/science.1179047 20929839

[20] Cooper RL, Thiery AP, Fletcher AG, Delbarre DJ, Rasch LJ, Fraser GJ. An ancient Turing-like patterning mechanism regulates skin denticle development in sharks. Sci Adv. 2018;4(11):eaau5484. doi: 10.1126/sciadv.aau5484 30417097 PMC6221541

[21] Cooper RL, Lloyd VJ, Di-Poï N, Fletcher AG, Barrett PM, Fraser GJ. Conserved gene signalling and a derived patterning mechanism underlie the development of avian footpad scales. Evodevo. 2019;10:19. doi: 10.1186/s13227-019-0130-9 31428299 PMC6693258

[22] Milinkovitch MC, Jahanbakhsh E, Zakany S. The unreasonable effectiveness of reaction diffusion in vertebrate skin color patterning. Annu Rev Cell Dev Biol. 2023;39:145–74. doi: 10.1146/annurev-cellbio-120319-024414 37843926

[23] Tzika AC, Ullate-Agote A, Zakany S, Kummrow M, Milinkovitch MC. Somitic positional information guides self-organized patterning of snake scales. Sci Adv. 2023;9(24):eadf8834. doi: 10.1126/sciadv.adf8834 37315141 PMC10266723

[24] Shyer AE, Rodrigues AR, Schroeder GG, Kassianidou E, Kumar S, Harland RM. Emergent cellular self-organization and mechanosensation initiate follicle pattern in the avian skin. Science. 2017;357(6353):811–5. doi: 10.1126/science.aai7868 28705989 PMC5605277

[25] Painter KJ, Ho W, Headon DJ. A chemotaxis model of feather primordia pattern formation during avian development. J Theor Biol. 2018;437:225–38. doi: 10.1016/j.jtbi.2017.10.026 29097151

[26] Ho WKW, Freem L, Zhao D, Painter KJ, Woolley TE, Gaffney EA, et al. Feather arrays are patterned by interacting signalling and cell density waves. PLoS Biol. 2019;17(2):e3000132. doi: 10.1371/journal.pbio.3000132 30789897 PMC6383868

[27] Yue Z, Jiang T-X, Widelitz RB, Chuong C-M. Wnt3a gradient converts radial to bilateral feather symmetry via topological arrangement of epithelia. Proc Natl Acad Sci U S A. 2006;103(4):951–5. doi: 10.1073/pnas.0506894103 16418297 PMC1347975

[28] Cheng D, Yan X, Qiu G, Zhang J, Wang H, Feng T, et al. Contraction of basal filopodia controls periodic feather branching via Notch and FGF signaling. Nat Commun. 2018;9(1):1345. doi: 10.1038/s41467-018-03801-z 29632339 PMC5890251

[29] Groves I, Placzek M, Fletcher AG. Of mitogens and morphogens: modelling Sonic Hedgehog mechanisms in vertebrate development. Philos Trans R Soc Lond B Biol Sci. 2020;375(1809):20190660. doi: 10.1098/rstb.2019.0660 32829689 PMC7482217

[30] Dhouailly D. The avian ectodermal default competence to make feathers. Dev Biol. 2024;508:64–76. doi: 10.1016/j.ydbio.2024.01.002 38190932

[31] Stanton BZ, Peng LF. Small-molecule modulators of the Sonic Hedgehog signaling pathway. Mol Biosyst. 2010;6(1):44–54. doi: 10.1039/b910196a 20024066

[32] Jain S, Song R, Xie J. Sonidegib: mechanism of action, pharmacology, and clinical utility for advanced basal cell carcinomas. Onco Targets Ther. 2017;10:1645–53. doi: 10.2147/OTT.S130910 28352196 PMC5360396

[33] Carballo GB, Honorato JR, de Lopes GPF, Spohr TCL de SE. A highlight on Sonic hedgehog pathway. Cell Commun Signal. 2018;16(1):11. doi: 10.1186/s12964-018-0220-7 29558958 PMC5861627

[34] Yu M, Yue Z, Wu P, Wu D-Y, Mayer J-A, Medina M, et al. The biology of feather follicles. Int J Dev Biol. 2004;48(2–3):181–91. doi: 10.1387/ijdb.031776my 15272383 PMC4380223

[35] Cooper RL, Milinkovitch MC. Transient agonism of the sonic hedgehog pathway triggers a permanent transition of skin appendage fate in the chicken embryo. Sci Adv. 2023;9(20):eadg9619. doi: 10.1126/sciadv.adg9619 37196093 PMC10191425

[36] Ting-Berreth SA, Chuong C-M. Sonic hedgehog in feather morphogenesis: Induction of mesenchymal condensation and association with cell death. Dev Dyn. 1996;207(2):157–70. doi: 10.1002/(sici)1097-0177(199610)207:2<157::aid-aja4>3.0.co;2-g 8906419

[37] McKinnell IW, Turmaine M, Patel K. Sonic Hedgehog functions by localizing the region of proliferation in early developing feather buds. Dev Biol. 2004;272(1):76–88. doi: 10.1016/j.ydbio.2004.04.019 15242792

[38] Busby L, Aceituno C, McQueen C, Rich CA, Ros MA, Towers M. Sonic hedgehog specifies flight feather positional information in avian wings. Development. 2020;147(9):dev188821. doi: 10.1242/dev.188821 32376617 PMC7225127

[39] Cooper RL, Santos-Durán G, Milinkovitch MC. Protocol for the rapid intravenous in ovo injection of developing amniote embryos. STAR Protoc. 2023;4(2):102324. doi: 10.1016/j.xpro.2023.102324 37210721 PMC10209871

[40] Pan S, Wu X, Jiang J, Gao W, Wan Y, Cheng D, et al. Discovery of NVP-LDE225, a potent and selective smoothened antagonist. ACS Med Chem Lett. 2010;1(3):130–4. doi: 10.1021/ml1000307 24900187 PMC4007689

[41] Olsson JE, Kamachi Y, Penning S, Muscat GE, Kondoh H, Koopman P. Sox18 expression in blood vessels and feather buds during chicken embryogenesis. Gene. 2001;271(2):151–8. doi: 10.1016/s0378-1119(01)00505-4 11418236

[42] Wu P, Yan J, Lai Y-C, Ng CS, Li A, Jiang X, et al. Multiple regulatory modules are required for scale-to-feather conversion. Mol Biol Evol. 2018;35(2):417–30. doi: 10.1093/molbev/msx295 29177513 PMC5850302

[43] Gene Ontology Consortium, Aleksander SA, Balhoff J, Carbon S, Cherry JM, Drabkin HJ, et al. The gene ontology knowledgebase in 2023. Genetics. 2023;224(1):iyad031. doi: 10.1093/genetics/iyad031 36866529 PMC10158837

[44] Kanehisa M, Goto S. KEGG: kyoto encyclopedia of genes and genomes. Nucleic Acids Res. 2000;28(1):27–30. doi: 10.1093/nar/28.1.27 10592173 PMC102409

[45] Otsuka A, Levesque MP, Dummer R, Kabashima K. Hedgehog signaling in basal cell carcinoma. J Dermatol Sci. 2015;78(2):95–100. doi: 10.1016/j.jdermsci.2015.02.007 25766766

[46] Villani A, Fabbrocini G, Costa C, Scalvenzi M. Sonidegib: safety and efficacy in treatment of advanced basal cell carcinoma. Dermatol Ther (Heidelb). 2020;10(3):401–12. doi: 10.1007/s13555-020-00378-8 32297221 PMC7211768

[47] Renier N, Adams EL, Kirst C, Wu Z, Azevedo R, Kohl J, et al. Mapping of brain activity by automated volume analysis of immediate early genes. Cell. 2016;165(7):1789–802. doi: 10.1016/j.cell.2016.05.007 27238021 PMC4912438

[48] Kanehisa M, Furumichi M, Sato Y, Ishiguro-Watanabe M, Tanabe M. KEGG: integrating viruses and cellular organisms. Nucleic Acids Res. 2021;49(D1):D545–51. doi: 10.1093/nar/gkaa970 33125081 PMC7779016

[49] Ge SX, Jung D, Yao R. ShinyGO: a graphical gene-set enrichment tool for animals and plants. Bioinformatics. 2020;36(8):2628–9. doi: 10.1093/bioinformatics/btz931 31882993 PMC7178415

[50] Luo W, Brouwer C. Pathview: an R/Bioconductor package for pathway-based data integration and visualization. Bioinformatics. 2013;29(14):1830–1. doi: 10.1093/bioinformatics/btt285 23740750 PMC3702256

