# Supplementary material for: In vivo sonic hedgehog pathway antagonism temporarily results in ancestral proto-feather-like structures in the chicken
Source: PLoS Biol. 2025 Mar 20;23(3):e3003061. doi: 10.1371/journal.pbio.3003061 (PMC12136001; doi:10.1371/journal.pbio.3003061)
Supplement: S1 Fig — Samples fixed following treatment with sonidegib at E9 were fixed from E10 to E14. (A) DMSO control samples reveal normal outgrowth of feather buds. (B–D) Sonidegib-treated samples exhibit a dose-dependent effect, with stronger sonidegib doses corresponding to less developed feather buds by E14. (PDF) [file pbio.3003061.s001.pdf]

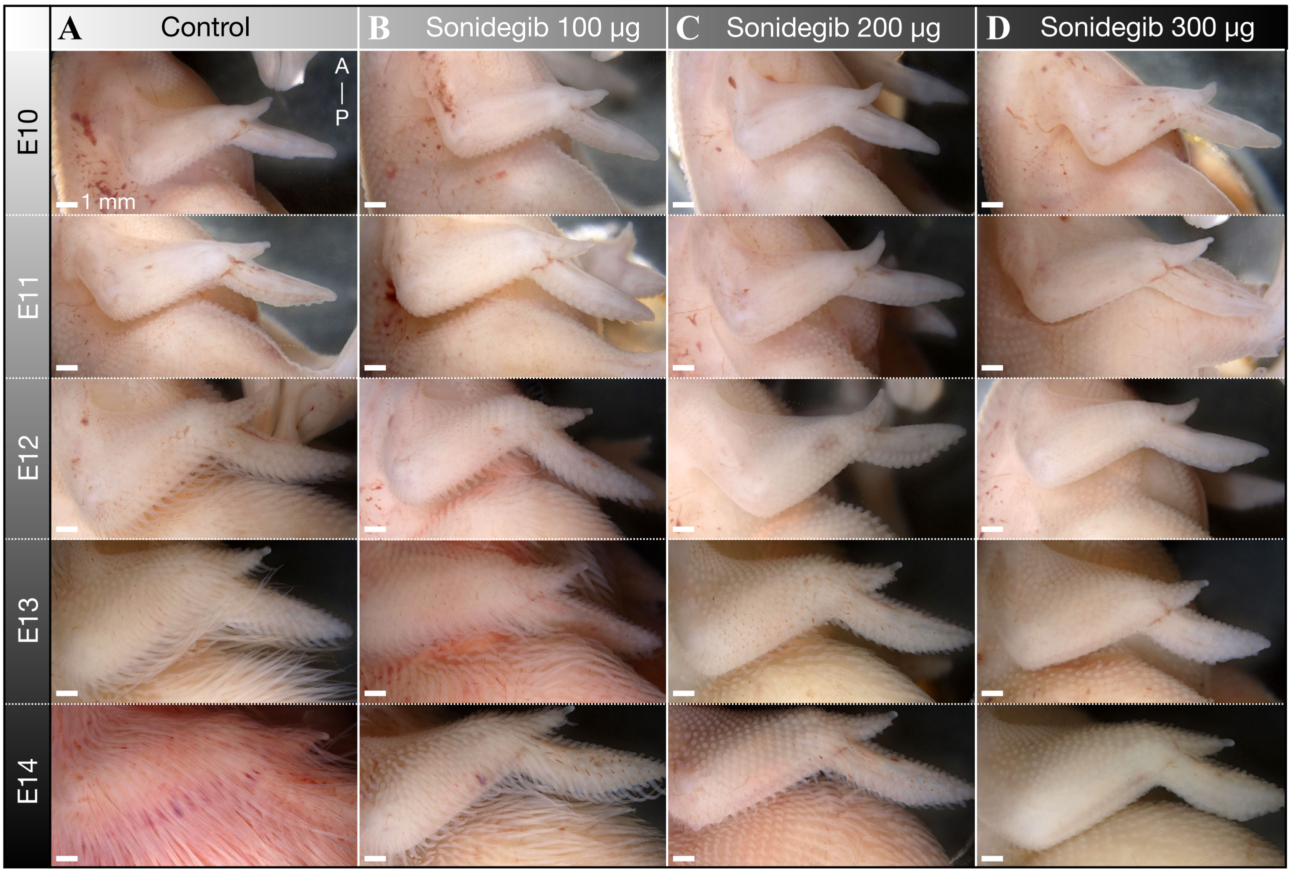

S1 Fig: Sonidegib treatment arrests feather morphogenesis. Samples fixed following treatment with sonidegib at E9 were fixed from E10 to E14. **(A)** DMSO control samples reveal normal outgrowth of feather buds. (**B-D**) Sonidegib-treated samples exhibit a dose-dependent effect, with stronger sonidegib doses corresponding to less developed feather buds by E14.
