# Supplementary material for: In vivo sonic hedgehog pathway antagonism temporarily results in ancestral proto-feather-like structures in the chicken
Source: PLoS Biol. 2025 Mar 20;23(3):e3003061. doi: 10.1371/journal.pbio.3003061 (PMC12136001; doi:10.1371/journal.pbio.3003061)
Supplement: S6 Fig — Chicken embryos injected at E9 with either DMSO (controls) or sonidegib (100, 200, or 300 μg) are shown. (PDF) [file pbio.3003061.s006.pdf]

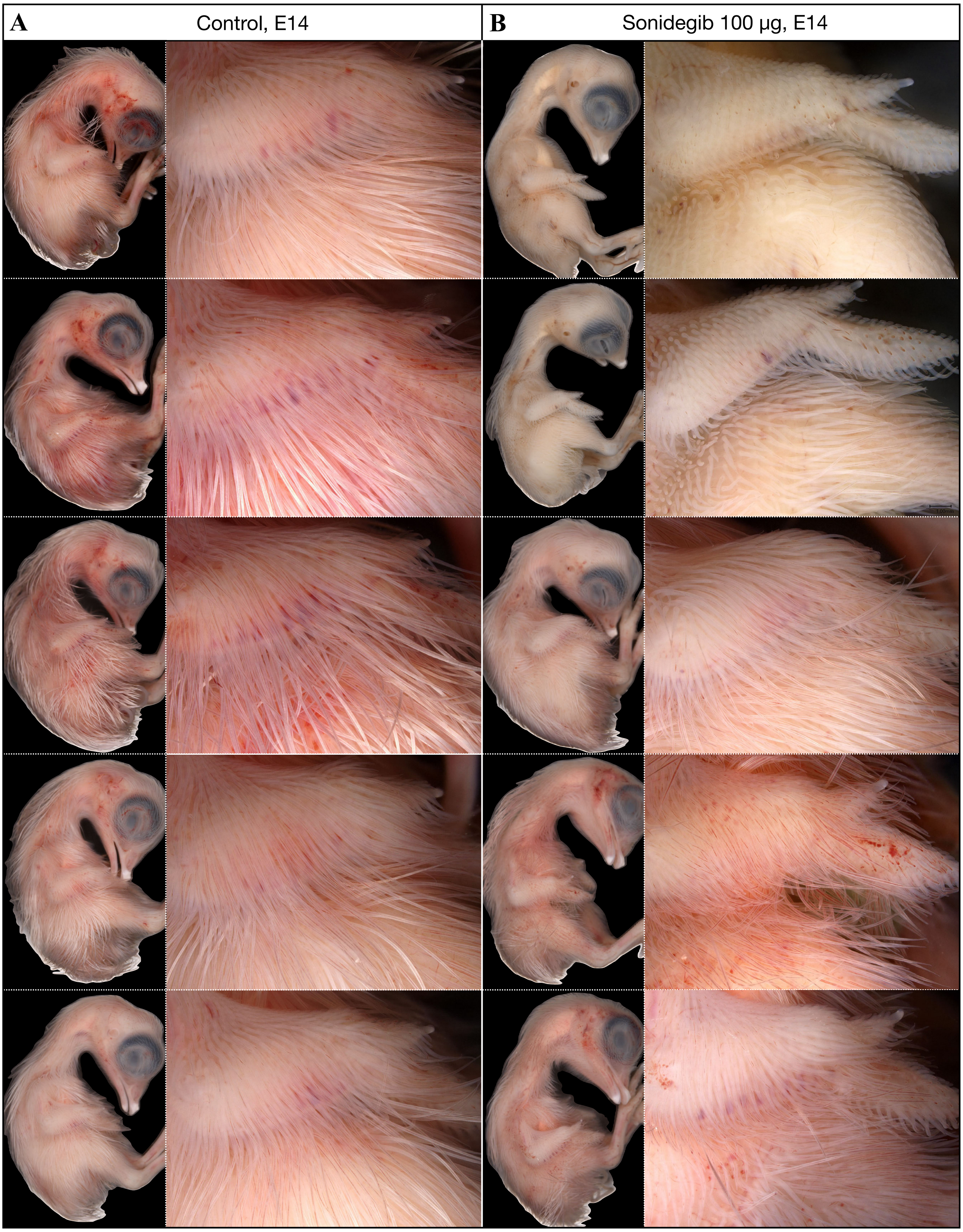

S6 Fig: Experimental replicates from sonidegib treatments at E14. Chicken embryos injected at E9 with either DMSO (controls) or sonidegib (100, 200, or 300 μg) are shown.

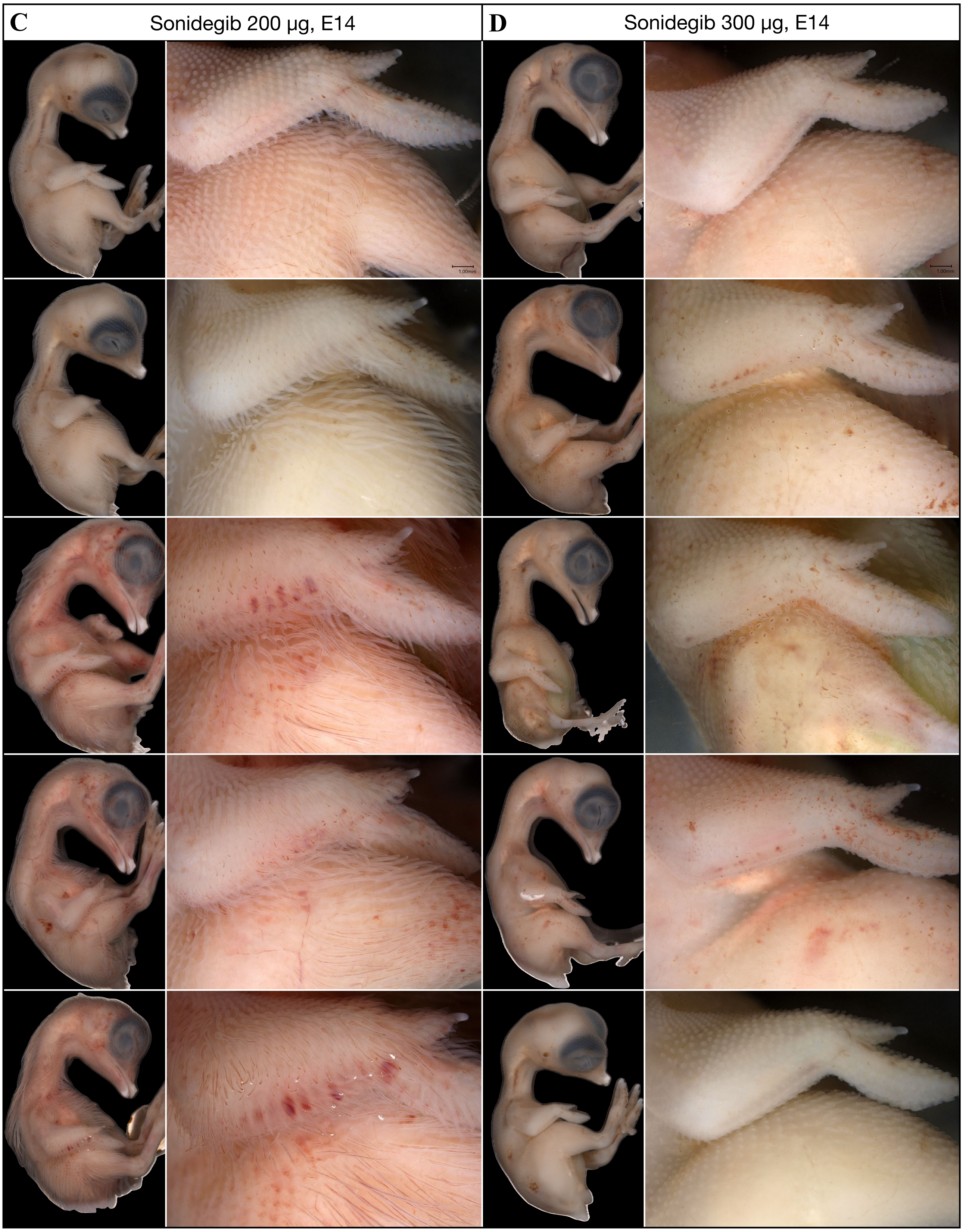

S6 Fig (continued).
