# Supplementary material for: In vivo sonic hedgehog pathway antagonism temporarily results in ancestral proto-feather-like structures in the chicken
Source: PLoS Biol. 2025 Mar 20;23(3):e3003061. doi: 10.1371/journal.pbio.3003061 (PMC12136001; doi:10.1371/journal.pbio.3003061)
Supplement: S7 Fig — Chicken embryos injected at E9 with either DMSO (controls) or sonidegib (100, 200, or 300 μg) are shown. Blue arrows show naked regions of the skin. (PDF) [file pbio.3003061.s007.pdf]

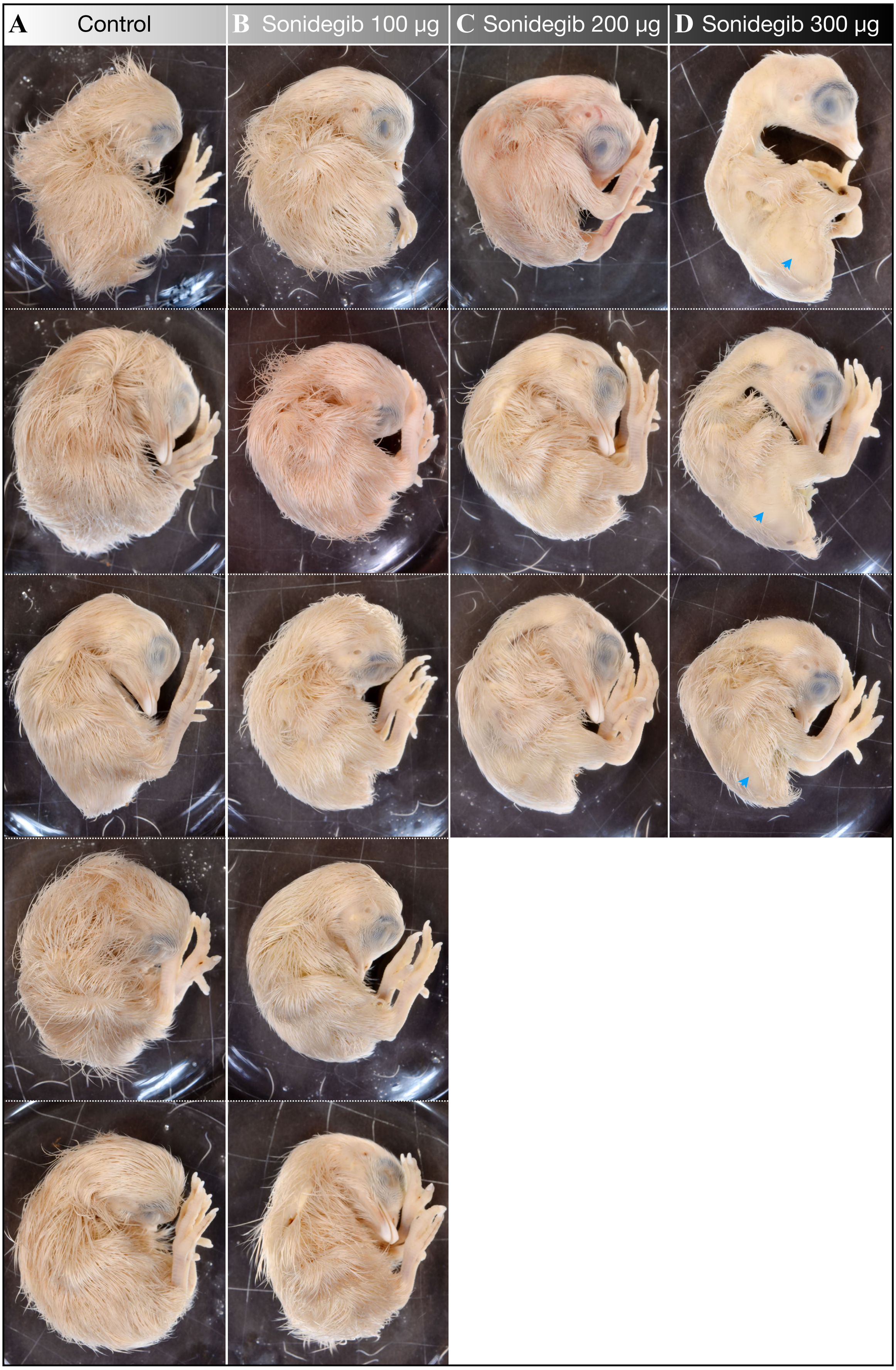

S7 Fig: Experimental replicates from sonidegib treatments at E17. Chicken embryos injected at E9 with either DMSO (controls) or sonidegib (100, 200, or 300 µg) are shown. Blue arrows show naked regions of the skin.
