# Supplementary material for: In vivo sonic hedgehog pathway antagonism temporarily results in ancestral proto-feather-like structures in the chicken
Source: PLoS Biol. 2025 Mar 20;23(3):e3003061. doi: 10.1371/journal.pbio.3003061 (PMC12136001; doi:10.1371/journal.pbio.3003061)
Supplement: S9 Fig — The clustering of individual RNA-seq sample replicates was undertaken for all sonidegib-treated and control samples at four developmental stages, using Euclidean distance and complete linkage of clusters. After filtering differentially expressed genes (DEGs) with a false-discovery rate (FDR) adjusted P-value of ≤0.05, the corresponding heat maps show that, at each time point, individual replicates cluster together according to their treatment type. See file S1 Data for the data underlying the graphs shown in the figure. (PDF) [file pbio.3003061.s009.pdf]

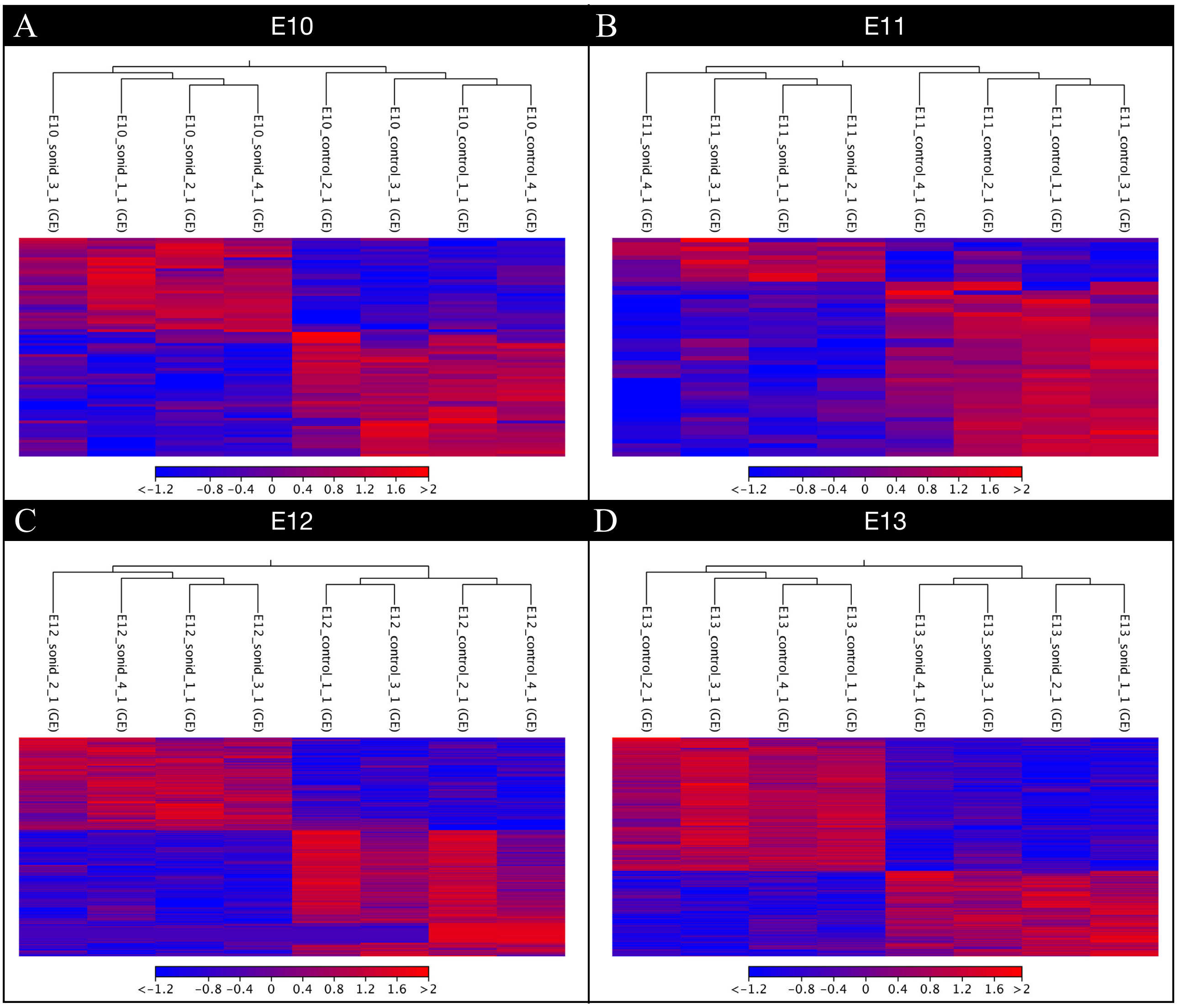

S9 Fig: RNA-seq sample clustering at different developmental time points. The clustering of individual RNA-seq sample replicates was undertaken for all sonidegib-treated and control samples at four developmental stages, using Euclidean distance and complete linkage of clusters. After filtering differentially expressed genes (DEGs) with a false-discovery rate (FDR) adjusted P value of ≤ 0.05, the corresponding heat maps show that, at each time point, individual replicates cluster together according to their treatment type. See file S1 for individual numerical values.
