# Supplementary material for: In vivo sonic hedgehog pathway antagonism temporarily results in ancestral proto-feather-like structures in the chicken
Source: PLoS Biol. 2025 Mar 20;23(3):e3003061. doi: 10.1371/journal.pbio.3003061 (PMC12136001; doi:10.1371/journal.pbio.3003061)
Supplement: S10 Fig — Individual genes are only shown if they exhibit differential expression (false-discovery rate adjusted P-value of ≤0.05) at a minimum of one embryonic stage, with the exceptions of Gli2 and Smo. Significant differential expression is shown with a yellow outline. (A) Multiple members of the Shh pathway are continuously down-regulated. Shh itself is down-regulated from E11 onwards. (B, C) Other skin appendage-associated genes are down-regulated at later developmental stages, indicative of their role in feather morphogenesis which is perturbed in treated samples. (D) We also observe dramatic down-regulation in genes associated with keratin production due to the absence of advanced feather bud morphogenesis in sonidegib-treated samples. See file S1 Data for the data underlying the graphs shown in the figure. (PDF) [file pbio.3003061.s010.pdf]

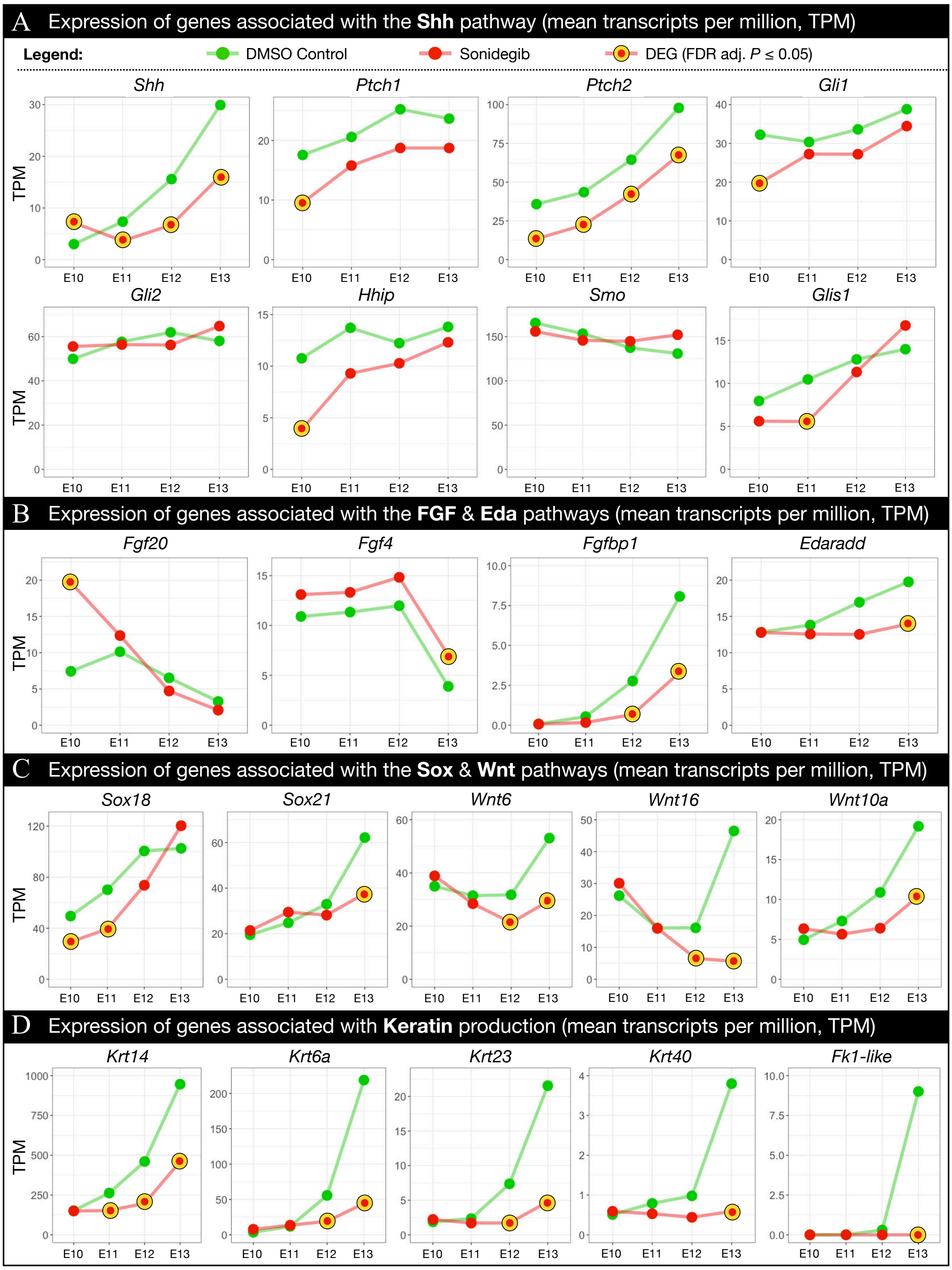

S10 Fig: Temporal changes in gene expression (RNA-seq. data; transcripts per million, TPM) after sonidegib treatment. Individual genes are only shown if they exhibit differential expression (false discovery rate adjusted P value of ≤ 0.05) at a minimum of one embryonic stage, with the exceptions of *Gli2* and *Smo*. Significant differential expression is shown with a yellow outline. **(A)** Multiple members of the Shh pathway are continuously down-regulated. *Shh* itself is down-regulated from E11 onwards. (**B-C**) Other skin appendage-associated genes are down-regulated at later developmental stages, indicative of their role in feather morphogenesis which is perturbed in treated samples. **(D)** We also observe dramatic down-regulation in genes associated with keratin production due to the absence of advanced feather bud morphogenesis in sonidegib-treated samples. See file S1 for individual numerical expression values for all replicates.
