# Supplementary material for: In vivo sonic hedgehog pathway antagonism temporarily results in ancestral proto-feather-like structures in the chicken
Source: PLoS Biol. 2025 Mar 20;23(3):e3003061. doi: 10.1371/journal.pbio.3003061 (PMC12136001; doi:10.1371/journal.pbio.3003061)
Supplement: S11 Fig — The 10 most significant GO terms (within “biological processes”) are reported for DEG sets comparing sonidegib-treated and control samples at each developmental stage. (A, B) Shh is associated with the 10 most significant GO terms at E10 and E11. Other Shh pathway members—including Ptch1, Ptch2, Gli1 and Glis1—are also frequently reported. (C) At E12, Shh is reported in 9 of the 10 most significant GO terms, and (D) at E13, Shh is reported in five of the 10 most significant GO terms. (PDF) [file pbio.3003061.s011.pdf]

**A** GO term (biological process) enrichment analysis of DGE at E10					
**GO term**	**Description**	**DE Genes**	**DE Genes (Names)**	**P-values**	**FDR p-value**
**10646**	regulation of cell communication	9	CRHBP, DLL1, FGF20, GPC3, PDE8B, PLK2, **PTCH1, PTCH2, SHH**	4.85E-03	0.69
**23051**	regulation of signaling	9	CRHBP, DLL1, FGF20, GPC3, PDE8B, PLK2, **PTCH1, PTCH2, SHH**	4.85E-03	0.69
**9966**	regulation of signal transduction	8	CRHBP, DLL1, FGF20, GPC3, PDE8B, **PTCH1**, **PTCH2**, **SHH**	0.01	0.69
**48518**	positive regulation of biological process	8	CYTL1, FGF20, **GLI1,** GPC3, PDE8B, **SHH**, SOX18, TFAP2C	0.01	0.69
**48522**	positive regulation of cellular process	8	CYTL1, FGF20, **GLI1,** GPC3, PDE8B, **SHH**, SOX18, TFAP2C	0.01	0.69
**48583**	regulation of response to stimulus	8	CRHBP, DLL1, FGF20, GPC3, PDE8B, **PTCH1, PTCH2, SHH**	0.01	0.69
**9893**	positive regulation of metabolic process	7	CYTL1, FGF20, **GLI1**, PDE8B, **SHH**, SOX18, TFAP2C	0.02	0.75
**10604**	positive regulation of macromolecule metabolic process	7	CYTL1, FGF20, **GLI1**, PDE8B, **SHH**, SOX18, TFAP2C	0.02	0.75
**31323**	regulation of cellular metabolic process	7	CYTL1, FGF20, **GLI1**, PDE8B, **SHH**, SOX18, TFAP2C	0.02	0.75
**31325**	positive regulation of cellular metabolic process	7	CYTL1, FGF20, **GLI1**, PDE8B, **SHH**, SOX18, TFAP2C	0.02	0.75

**B** GO term (biological process) enrichment analysis of DGE at E11					
**GO term**	**Description**	**DE Genes**	**DE Genes (Names)**	**P-values**	**FDR p-value**
**10646**	regulation of cell communication	6	CALB2, GATA3, GPC3, PLK2, **PTCH2, SHH**	0.05	0.76
**23051**	regulation of signaling	6	CALB2, GATA3, GPC3, PLK2, **PTCH2, SHH**	0.05	0.76
**9987**	cellular process	11	CALB2, COL10A1, DLX3, GATA3, GCH1, GPC3, KRT14, PLK2, PRDM1, **SHH**, SOX18	0.13	0.76
**9889**	regulation of biosynthetic process	9	DLX3, GATA3, GCH1, **GLIS1**, PRDM1, **SHH**, SOX18, TCF7, TFAP2C	0.14	0.76
**19222**	regulation of metabolic process	9	DLX3, GATA3, GCH1, **GLIS1**, PRDM1, **SHH**, SOX18, TCF7, TFAP2C	0.14	0.76
**31323**	regulation of cellular metabolic process	9	DLX3, GATA3, GCH1, **GLIS1**, PRDM1, **SHH**, SOX18, TCF7, TFAP2C	0.14	0.76
**31326**	regulation of cellular biosynthetic process	9	DLX3, GATA3, GCH1, **GLIS1**, PRDM1, **SHH**, SOX18, TCF7, TFAP2C	0.14	0.76
**51171**	regulation of nitrogen compound metabolic process	9	DLX3, GATA3, GCH1, **GLIS1**, PRDM1, **SHH**, SOX18, TCF7, TFAP2C	0.14	0.76
**9966**	regulation of signal transduction	4	GATA3, GPC3, **PTCH2, SHH**	0.16	0.76
**48583**	regulation of response to stimulus	4	GATA3, GPC3, **PTCH2, SHH**	0.16	0.76

S11 Fig: GO term enrichment analyses of differentially expressed gene sets at four developmental time points. The ten most significant GO terms (within 'biological processes') are reported for DEG sets comparing sonidegib-treated and control samples at each developmental stage. (**A-B**) *Shh* is associated with the ten most significant GO terms at E10 and E11. Other *Shh* pathway members — including *Ptch1*, *Ptch2*, *Gli1* and *Glis1* — are also frequently reported. (**C**) At E12, *Shh* is reported in 9 of the ten most significant GO terms, and (**D**) at E13, *Shh* is reported in five of the ten most significant GO terms.

C	GO term (biological process) enrichment analysis of DGE at E12				
**GO term**	**Description**	**DE Genes**	**DE Genes (Names)**	**P-values**	**FDR p-value**
**45595**	regulation of cell differentiation	5	CEBPB, GATA3, MUSK, RUNX3, **SHH**	0.05	0.91
**50793**	regulation of developmental process	5	CEBPB, GATA3, MUSK, RUNX3, **SHH**	0.05	0.91
**9893**	positive regulation of metabolic process	7	CEBPB, GATA3, GCH1, **SHH**, TFAP2B, TFAP2C, ZNF750	0.06	0.91
**31325**	positive regulation of cellular metabolic process	7	CEBPB, GATA3, GCH1, **SHH**, TFAP2B, TFAP2C, ZNF750	0.06	0.91
**7010**	cytoskeleton organization	12	FAM83H, KRT14, KRT17, KRT18, KRT23, KRT5, KRT6A, KRT80, LMOD3, TMOD1, TNNT3, TRIM36	0.08	0.91
**48518**	positive regulation of biological process	12	CEBPB, FAM83H, GATA3, GCH1, GPC3, KITLG, MUSK, PAWR, **SHH**, TFAP2B, TFAP2C, ZNF750	0.08	0.91
**48522**	positive regulation of cellular process	12	CEBPB, FAM83H, GATA3, GCH1, GPC3, KITLG, MUSK, PAWR, **SHH**, TFAP2B, TFAP2C, ZNF750	0.08	0.91
**9967**	positive regulation of signal transduction	4	GATA3, GPC3, MUSK, **SHH**	0.09	0.91
**10647**	positive regulation of cell communication	4	GATA3, GPC3, MUSK, **SHH**	0.09	0.91
**23056**	positive regulation of signaling	4	GATA3, GPC3, MUSK, **SHH**	0.09	0.91

D	GO term (biological process) enrichment analysis of DGE at E13				
**GO term**	**Description**	**DE Genes**	**DE Genes (Names)**	**P-values**	**FDR p-value**
**10468**	regulation of gene expression	56	BHLHE40, CCDC160, CEBPB, CSRNP3, DACH1, DLX3, DLX4, DLX5, EAF2, EGR1, EGR3, EHF, ELF5, ESRP1, ESRP2, FAM83G, FOSL2, FOXN1, GATA3, GRHL3, HOXC9, IRF6, JUP, KCTD1, KLF5, KROX20, LBX1, LMX1B, MSANTD1, MSC, MSX2, MYB, MYOCD, MYOD1, NDP, NFKBIZ, PITX1, PIWIL1, PLEKHN1, POU3F1, PPARG, RBM24, RUNX3, **SHH**, SOX21, SP6, TCF7, TFAP2A, TFAP2B, TFAP2C, TFAP2E, TFCP2L1, TP63, TWIST3, VGLL2, ZNF750	0.03	1.00
**51252**	regulation of RNA metabolic process	53	BHLHE40, CCDC160, CEBPB, CSRNP3, DACH1, DLX3, DLX4, DLX5, EAF2, EGR1, EGR3, EHF, ELF5, ESRP1, ESRP2, FAM83G, FOSL2, FOXN1, GATA3, GRHL3, HOXC9, IRF6, JUP, KCTD1, KLF5, KROX20, LBX1, LMX1B, MSANTD1, MSC, MSX2, MYB, MYOCD, MYOD1, NDP, PITX1, POU3F1, PPARG, RBM24, RUNX3, **SHH**, SOX21, SP6, TCF7, TFAP2A, TFAP2B, TFAP2C, TFAP2E, TFCP2L1, TP63, TWIST3, VGLL2, ZNF750	0.03	1.00
**3012**	muscle system process	12	CACNA1S, LMOD3, MYH1B, MYH1C, TMOD1, TNNC1, TNNC2, TNNI1, TNNI2, TNNT2, TNNT3, TPM2	0.04	1.00
**6936**	muscle contraction	12	CACNA1S, LMOD3, MYH1B, MYH1C, TMOD1, TNNC1, TNNC2, TNNI1, TNNI2, TNNT2, TNNT3, TPM2	0.04	1.00
**45103**	intermediate filament-based process	12	DES, DSP, FAM83H, KRT14, KRT17, KRT18, KRT23, KRT5, KRT6A, KRT7, KRT80, PPL	0.04	1.00
**45104**	intermediate filament cytoskeleton organization	12	DES, DSP, FAM83H, KRT14, KRT17, KRT18, KRT23, KRT5, KRT6A, KRT7, KRT80, PPL	0.04	1.00
**19222**	regulation of metabolic process	66	BHLHE40, CCDC160, CEBPB, CSRNP3, DACH1, DLX3, DLX4, DLX5, DUSP5, EAF2, EGR1, EGR3, EHF, ELF5, EPGN, ESRP1, ESRP2, FAM83G, FGF4, FOSL2, FOXN1, GATA3, GCH1, GPR37, GRHL3, HIP1R, HOXC9, IRF6, JUP, KCTD1, KLF5, KLHL31, KLHL40, KROX20, LBX1, LMX1B, MSANTD1, MSC, MSX2, MYB, MYOCD, MYOD1, NDP, NFKBIZ, PITX1, PIWIL1, PLEKHN1, POU3F1, PPARG, RBM24, RUNX3, SERPINB5, **SHH**, SOX21, SP6, TCF7, TFAP2A, TFAP2B, TFAP2C, TFAP2E, TFCP2L1, TP63, TRIB1, TWIST3, VGLL2, ZNF750	0.04	1.00
**6357**	regulation of transcription from RNA polymerase II promoter	46	BHLHE40, CCDC160, CEBPB, CSRNP3, DACH1, DLX3, DLX4, DLX5, EGR1, EGR3, EHF, ELF5, FAM83G, FOSL2, FOXN1, GATA3, GRHL3, HOXC9, IRF6, JUP, KLF5, KROX20, LBX1, LMX1B, MSC, MSX2, MYB, MYOCD, MYOD1, PITX1, POU3F1, PPARG, RUNX3, **SHH**, SOX21, SP6, TCF7, TFAP2A, TFAP2B, TFAP2C, TFAP2E, TFCP2L1, TP63, TWIST3, VGLL2, ZNF750	0.05	1.00
**51171**	regulation of nitrogen compound metabolic process	56	BHLHE40, CCDC160, CEBPB, CSRNP3, DACH1, DLX3, DLX4, DLX5, EAF2, EGR1, EGR3, EHF, ELF5, ESRP1, ESRP2, FAM83G, FOSL2, FOXN1, GATA3, GCH1, GRHL3, HOXC9, IRF6, JUP, KCTD1, KLF5, KROX20, LBX1, LMX1B, MSANTD1, MSC, MSX2, MYB, MYOCD, MYOD1, NDP, PITX1, PIWIL1, PLEKHN1, POU3F1, PPARG, RBM24, RUNX3, **SHH**, SOX21, SP6, TCF7, TFAP2A, TFAP2B, TFAP2C, TFAP2E, TFCP2L1, TP63, TWIST3, VGLL2, ZNF750	0.05	1.00
**3008**	system process	15	ACTC1, CACNA1S, LMOD3, MYH1B, MYH1C, PENK, TMOD1, TNNC1, TNNC2, TNNI1, TNNI2, TNNT2, TNNT3, TPM2, UNC119	0.05	1.00

S11 Fig (continued).
