# Supplementary material for: In vivo sonic hedgehog pathway antagonism temporarily results in ancestral proto-feather-like structures in the chicken
Source: PLoS Biol. 2025 Mar 20;23(3):e3003061. doi: 10.1371/journal.pbio.3003061 (PMC12136001; doi:10.1371/journal.pbio.3003061)
Supplement: S12 Fig — KEGG pathway analysis of DEG sets comparing sonidegib-treated and control samples was used to identify key pathways at each time point. (A) The most significant detected KEGG pathways for each stage are listed. (B) At E10, “Hedgehog signaling” is the most significant KEGG pathway (individual DEGs present in our dataset are shown in red). (B–E) From E10 to E13, the most consistently detected significant KEGG pathway is ‘Basal cell carcinoma’, which is mediated by interactions of both Shh and Ptch. (PDF) [file pbio.3003061.s012.pdf]

A Stage	Most significant detected KEGG pathways
E10	Hedgehog signalling pathway, basal cell carcinoma, proteoglycans in cancer
E11	Basal cell carcinoma
E12	Basal cell carcinoma, melanogenesis, fatty acid elongation
E13	Basal cell carcinoma, melanogenesis, fatty acid elongation

B **E10: Hedgehog signalling pathway**

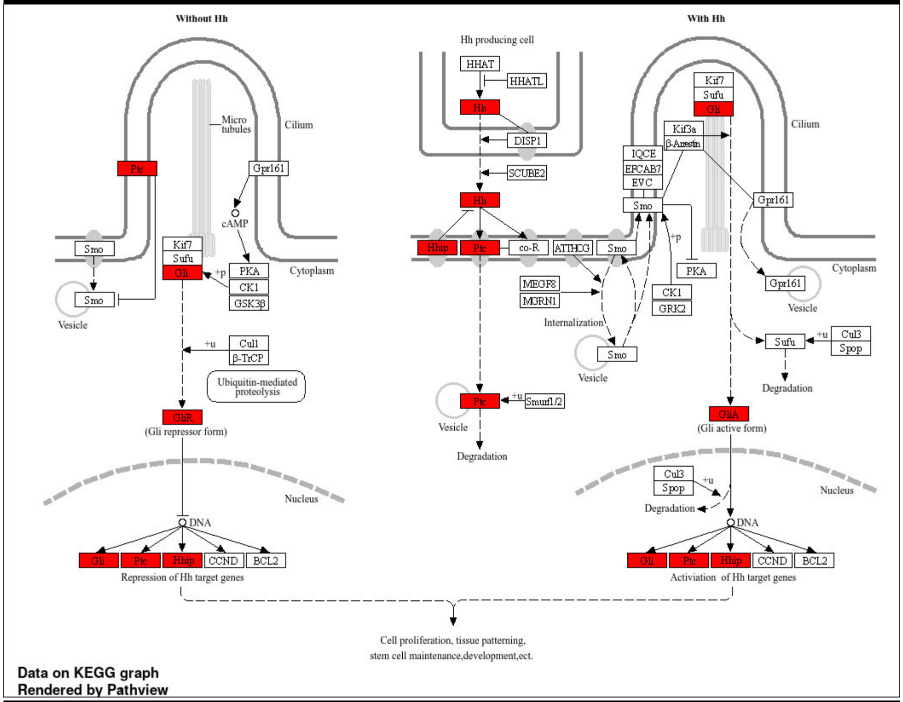

C **E11: Basal cell carcinoma**

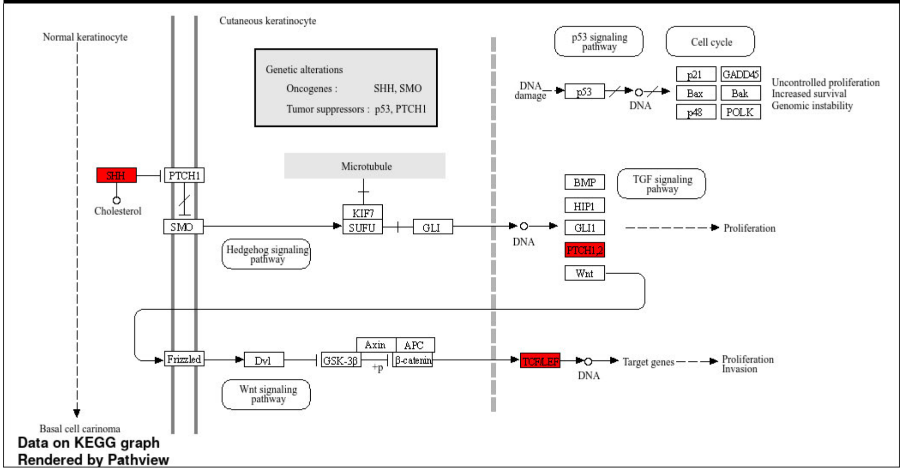

S12 Fig: Most significant KEGG pathways detected from DEG sets at four developmental time points. KEGG pathway analysis of DEG sets comparing sonidegib-treated and control samples was used to identify key pathways at each time point. (**A**) The most significant detected KEGG pathways for each stage are listed. (**B**) At E10, 'Hedgehog signalling' is the most significant KEGG pathway (individual DEGs present in our dataset are shown in red). (**B-E**) From E10 to E13, the most consistently detected significant KEGG pathway is 'Basal cell carcinoma', which is mediated by interactions of both *Shh* and *Ptch*.

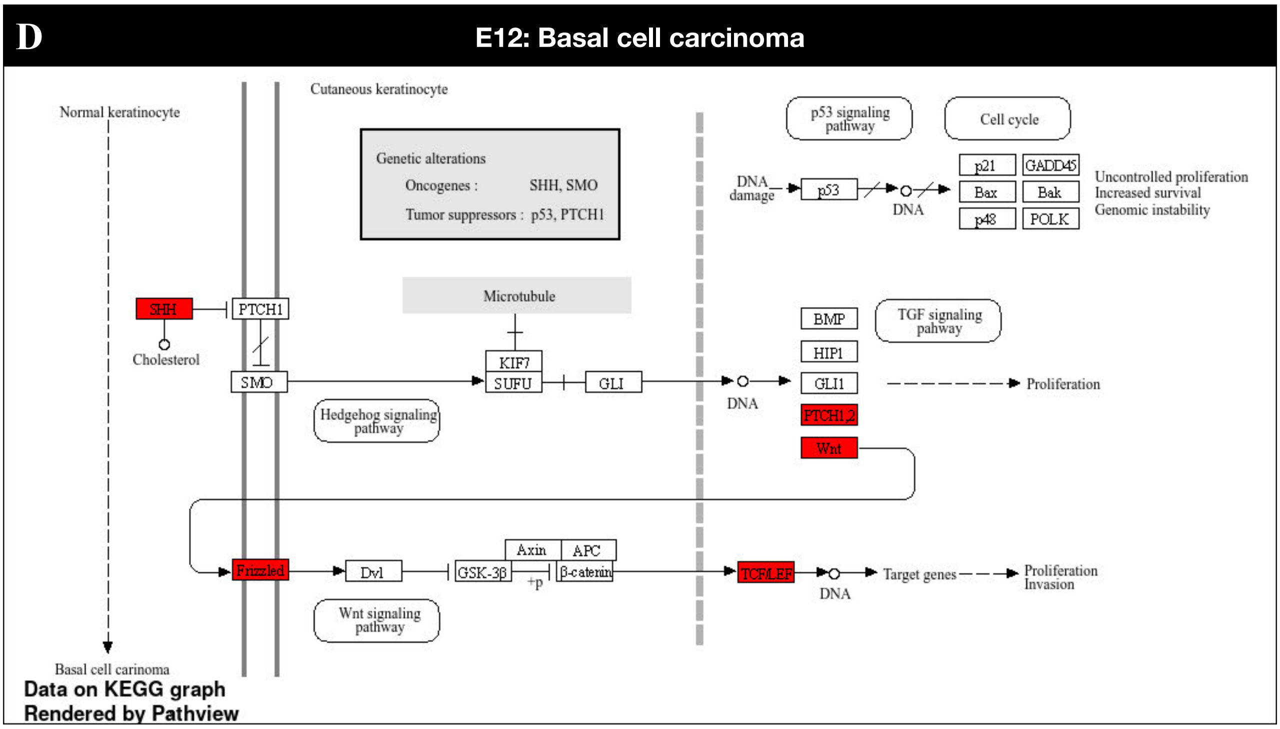

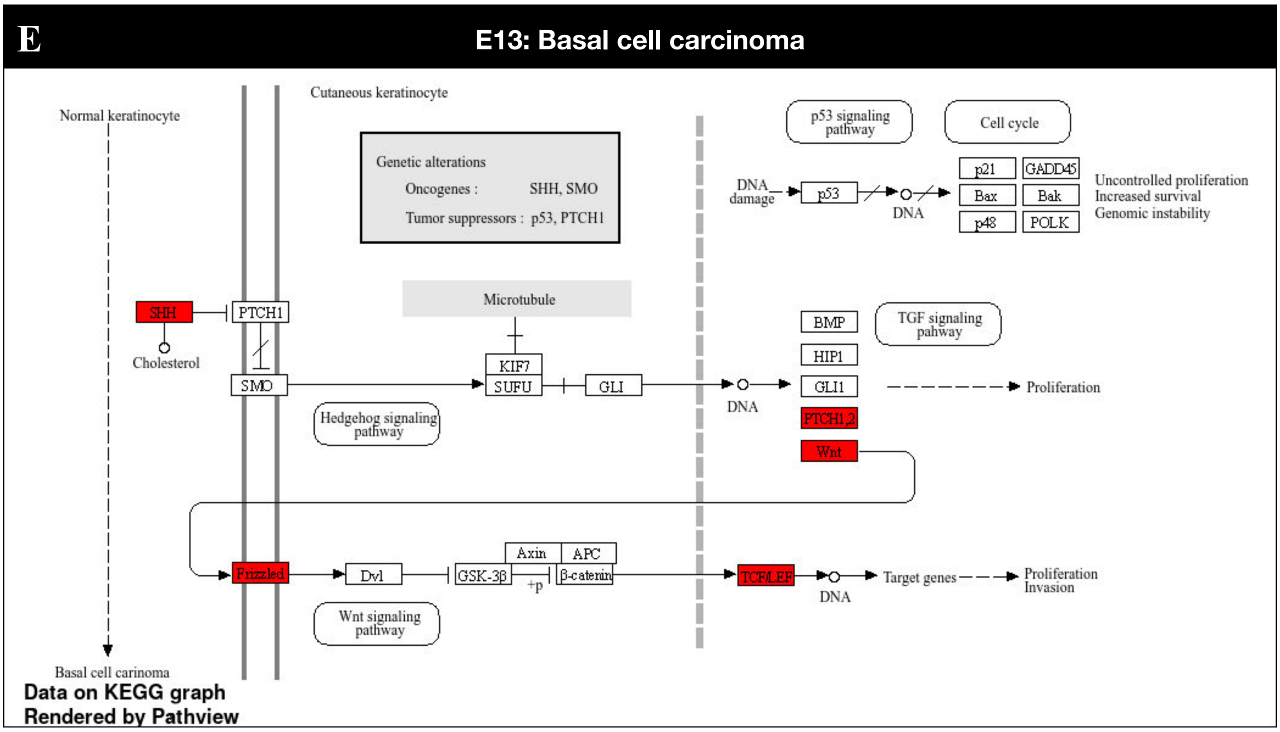

S12 Fig (continued).
