# Supplementary material for: In vivo sonic hedgehog pathway antagonism temporarily results in ancestral proto-feather-like structures in the chicken
Source: PLoS Biol. 2025 Mar 20;23(3):e3003061. doi: 10.1371/journal.pbio.3003061 (PMC12136001; doi:10.1371/journal.pbio.3003061)
Supplement: S13 Fig — Chicken embryos were treated with either (A) DMSO at E9 as a control, or (B) 300 μg sonidegib at E9, or (C) both 300 μg sonidegib at E9 and 50 μg SAG at E9, or (D) both 300 μg sonidegib at E9 and 100 μg SAG at E10, or (E) both 300 μg sonidegib at E9 and 150 μg SAG at E11. All samples were fixed and imaged at E14. (PDF) [file pbio.3003061.s013.pdf]

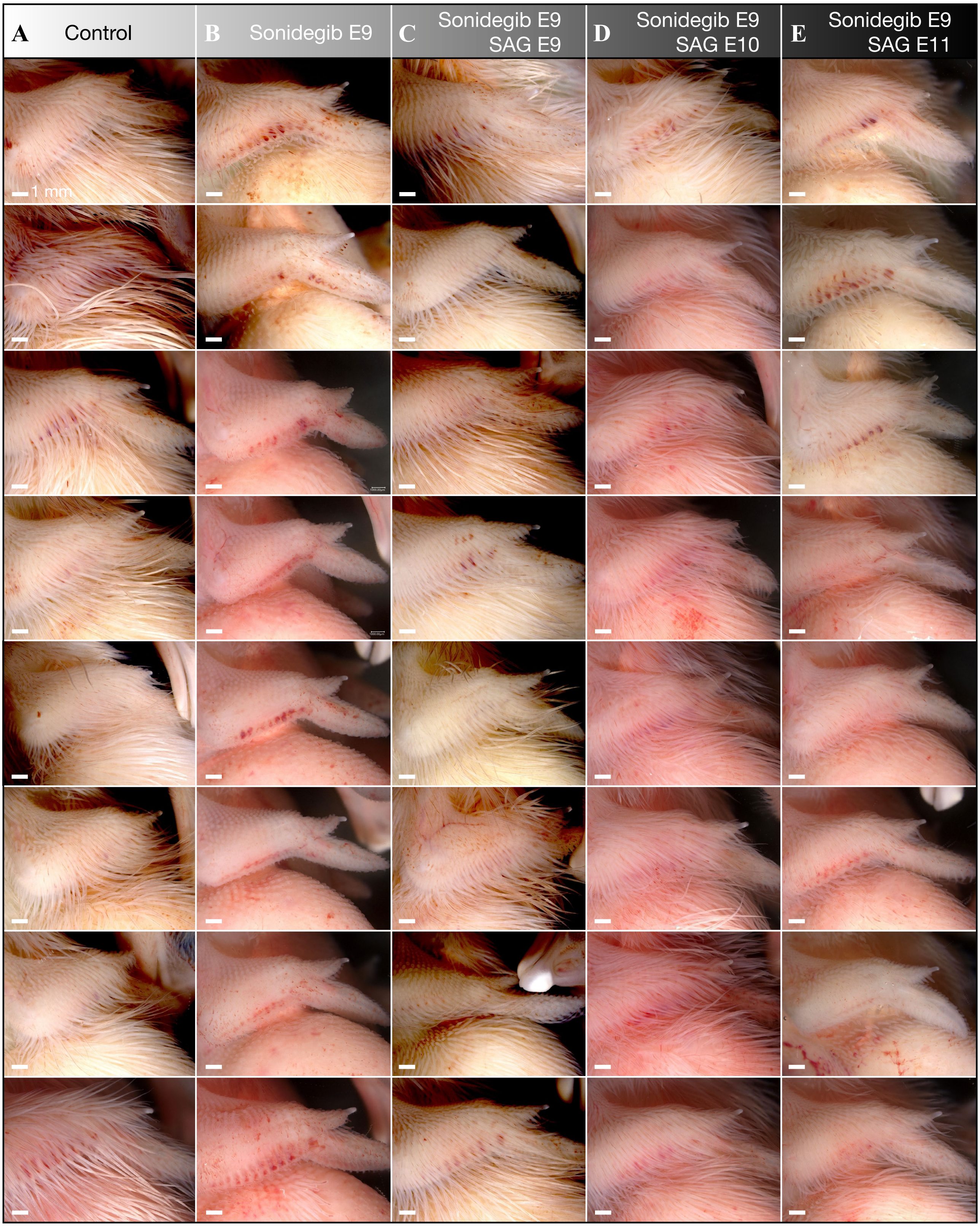

S13 Fig: Experimental replicates from combined sonidegib and SAG treatments. Chicken embryos were treated with either (**A**) DMSO at E9 as a control, or (**B**) 300 µg sonidegib at E9, or (**C**) both 300 µg sonidegib at E9 and 50 µg SAG at E9, or (**D**) both 300 µg sonidegib at E9 and 100 µg SAG at E10, or (**E**) both 300 µg sonidegib at E9 and 150 µg SAG at E11. All samples were fixed and imaged at E14.
