# Supplementary material for: In vivo sonic hedgehog pathway antagonism temporarily results in ancestral proto-feather-like structures in the chicken
Source: PLoS Biol. 2025 Mar 20;23(3):e3003061. doi: 10.1371/journal.pbio.3003061 (PMC12136001; doi:10.1371/journal.pbio.3003061)
Supplement: S14 Fig — Removing down-type feathers from the dorsal midline of control and sonidegib-treated chickens reveals a dose-dependent decrease in feather size resulting from treatments of increasing strength. (PDF) [file pbio.3003061.s014.pdf]

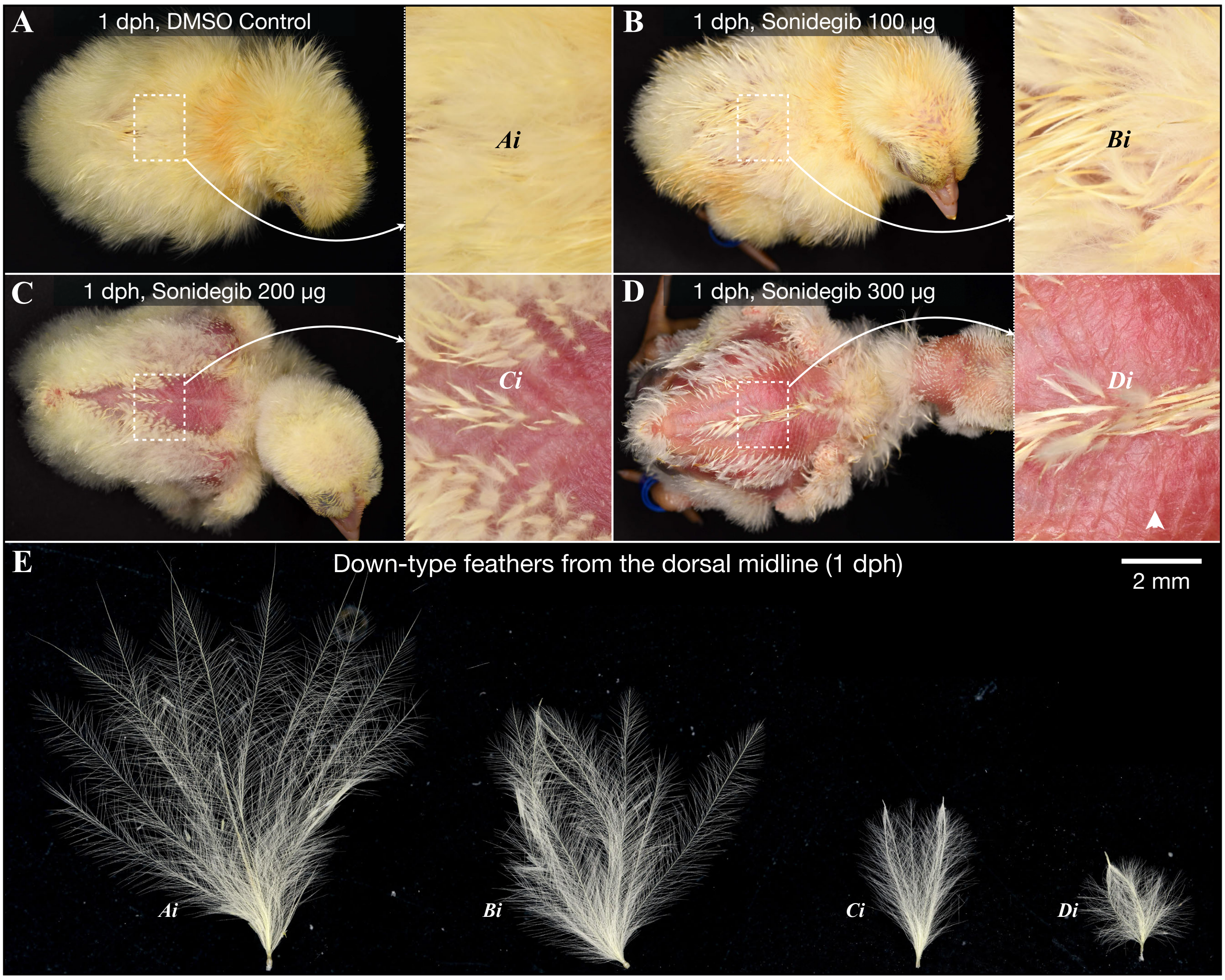

S14 Fig: Down-type feather morphology of hatched sonidegib-treated chickens at 1 dph. Removing down-type feathers from the dorsal midline of control and sonidegib-treated chickens reveals a dose-dependent decrease in feather size resulting from treatments of increasing strength.
