# Supplementary material for: In vivo sonic hedgehog pathway antagonism temporarily results in ancestral proto-feather-like structures in the chicken
Source: PLoS Biol. 2025 Mar 20;23(3):e3003061. doi: 10.1371/journal.pbio.3003061 (PMC12136001; doi:10.1371/journal.pbio.3003061)
Supplement: S15 Fig — Plucking the feathers from the bodies and wings of chickens injected at E9 with either DMSO (controls) or sonidegib (100, 200, or 300 μg) reveals no notable difference in the spatial distribution of feather follicles. (PDF) [file pbio.3003061.s015.pdf]

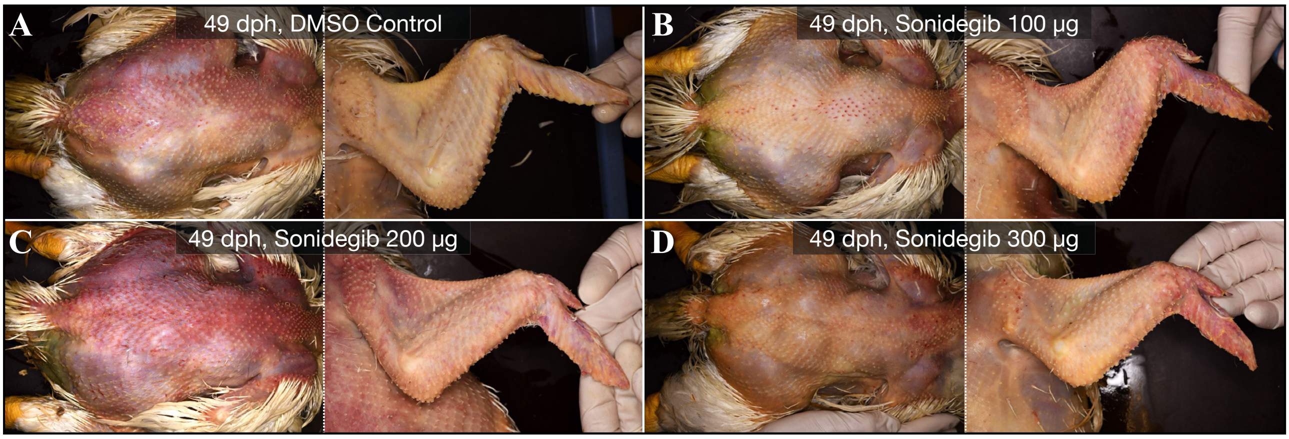

S15 Fig: Follicle patterning on the bodies and wings of sonidegib-treated chickens at 49 dph. Plucking the feathers from the bodies and wings of chickens injected at E9 with either DMSO (controls) or sonidegib (100, 200, or 300 μg) reveals no notable difference in the spatial distribution of feather follicles.
