# Supplementary material for: In vivo sonic hedgehog pathway antagonism temporarily results in ancestral proto-feather-like structures in the chicken
Source: PLoS Biol. 2025 Mar 20;23(3):e3003061. doi: 10.1371/journal.pbio.3003061 (PMC12136001; doi:10.1371/journal.pbio.3003061)
Supplement: S16 Fig — The wings of chickens injected at E9 with either DMSO (controls) or sonidegib (100, 200, or 300 μg) are shown from 1 dph to 49 dph. (A, B) Flight feathers can be observed emerging in both control and 100 μg sonidegib-treated samples at 1 dph (top row). These pennaceous feathers continue to develop until E49 (bottom row), at which stage they appear slightly smaller in samples treated with 100 μg sonidegib. (C, D) At 1 dph flight feathers are not visible on the wings of samples treated with 200 or 300 μg sonidegib. By 7 dph, some small and perturbed flight feathers emerge. However, by 49 dph, flight feathers are largely absent from both of these treatment groups, and the posterior region of the wing is instead covered with smaller contour feathers. (PDF) [file pbio.3003061.s016.pdf]

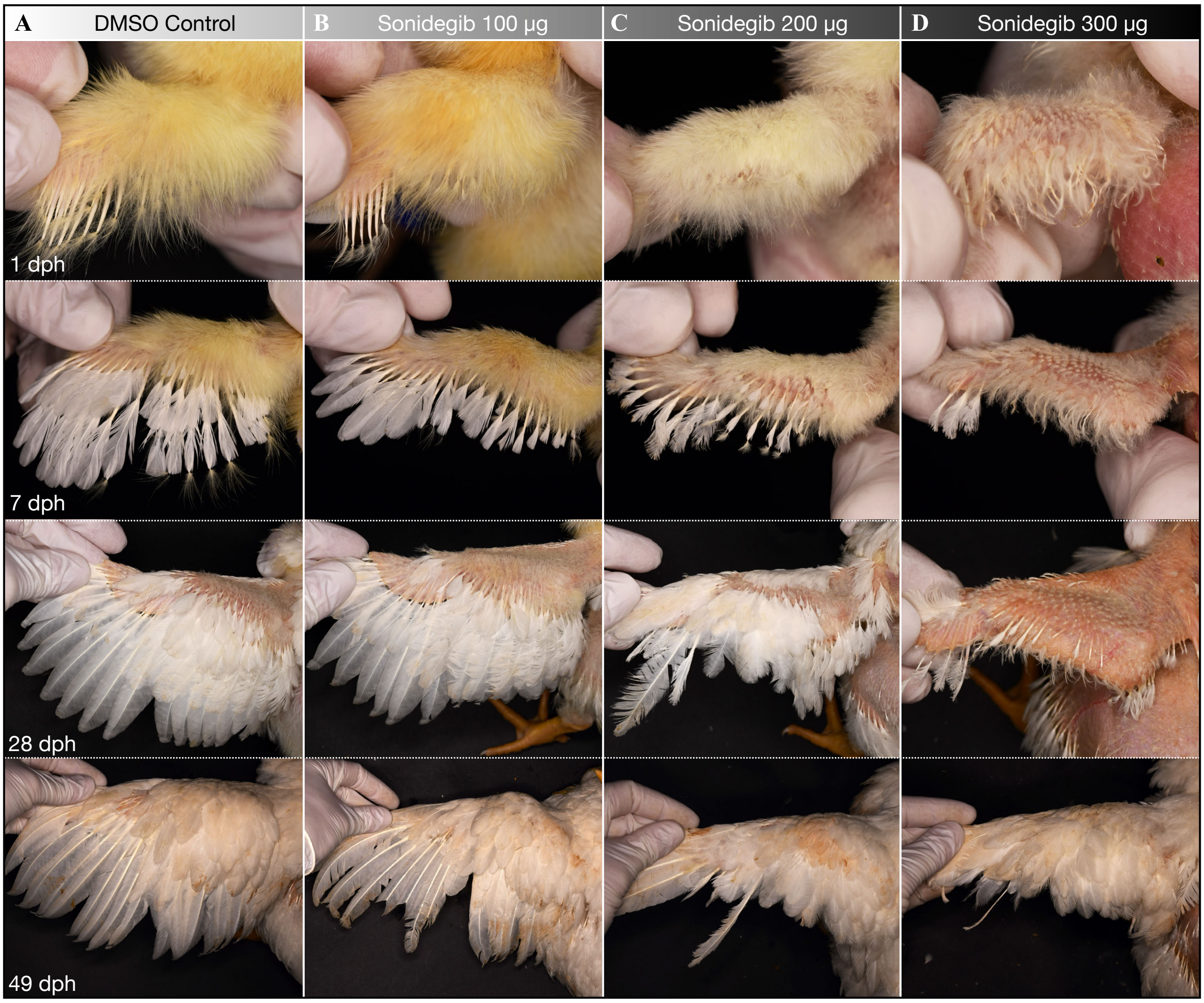

S16 Fig: The development of flight feathers is permanently reduced in sonidegib-treated chickens. The wings of chickens injected at E9 with either DMSO (controls) or sonidegib (100, 200, or 300 µg) are shown from 1 dph to 49 dph. **(A-B)** Flight feathers can be observed emerging in both control and 100 µg sonidegib-treated samples at 1 dph (top row). These pennaceous feathers continue to develop until E49 (bottom row), at which stage they appear slightly smaller in samples treated with 100 µg sonidegib. **(C-D)** At 1 dph flight feathers are not visible on the wings of samples treated with 200 or 300 µg sonidegib. By 7 dph, some small and perturbed flight feathers emerge. However, by 49 dph, flight feathers are largely absent from both of these treatment groups, and the posterior region of the wing is instead covered with smaller contour feathers.
